# Full-Field Dynamic Parameters and Tension Identification of Stayed Cables Using a Novel Holographic Vision-Based Method

**DOI:** 10.3390/s25164891

**Published:** 2025-08-08

**Authors:** Shuai Shao, Gang Liu, Zhongru Yu, Dongzhe Ren, Guojun Deng, Zhixiang Zhou

**Affiliations:** 1 School of Intelligent Information Engineering, Chongqing Aerospace Polytechnic, Chongqing 400021, China; shuai.shaos@outlook.com (S.S.); 13883164254@163.com (D.R.); 2 State Key Laboratory of Safety and Resilience of Civil Engineering in Mountain Area, Chongqing University, Chongqing 400044, China; 3 College of Civil Engineering, Southwest Jiaotong University, Chengdu 610031, China; zhongru_yu_swjtu@163.com; 4 China Merchants Chongqing Communications Technology Research & Design Institute Co., Ltd., Chongqing 400060, China; guojunforsea@gmail.com; 5 School of Civil and Transportation Engineering, Shenzhen University, Shenzhen 518060, China; zhixiangzhou@szu.edu.cn

**Keywords:** stayed cables, full-field dynamic parameters, cable tension, holographic vision-based method, optical flow tracking, motion amplification

## Abstract

**Highlights:**

**What are the main findings?**

- We propose a novel holographic vision-based method for full-field dynamic parameters and tension identification of stayed cables;
- We have proposed the first comprehensive, visual, and quantifiable strategy for stayed cable monitoring.

**What is the implication of the main finding?**

- The proposed HFPVM algorithm enhances the weak morphological motion information;
- The algorithm accurately identifies the natural frequencies, damping ratios, holographic modal shapes, and dynamic displacement vectors, especially in high-order modes.

**Abstract:**

Due to the slender geometry and low-amplitude vibrations of stayed cables, existing vision-based methods often fail to accurately identify their full-field dynamic parameters, especially the higher-order modes. This paper proposes a novel holographic vision-based method to accurately identify the high-order full-field dynamic parameters and estimate the tension of the stayed cables. Particularly, a full-field optical flow tracking algorithm is proposed to obtain the full-field dynamic displacement information of the stayed cable by tracking the changes in the optical flow field of the continuous motion signal spectral components of holographic feature points. Frequency-domain analysis is applied to extract the natural frequencies and damping ratios, and the vibration frequency method is used to estimate the tension. Additionally, an Eulerian-based amplification algorithm—holographic feature point video magnification (HFPVM)—is proposed for enhancing weak visual motion signals of the stayed cables, so that the morphological motion information of the stayed cables can be visualized. The effectiveness of the proposed method has been validated through experiments on the stayed cable models. Compared with the results obtained using contact sensors, the proposed holographic vision-based method can accurately identify the first five natural frequencies with overall errors below 5% and a maximum deviation of 6.86% in cable tension estimation. The first three normalized holographic mode shapes and dynamic displacement vectors are successfully identified, with the MAC value reaching up to 99.51%. This entirely non-contact vision-based method offers a convenient and low-cost approach for cable tension estimation, and this is also the first study to propose a comprehensive, visual, and quantifiable strategy for periodic or long-term monitoring of cable-supported structures, highlighting its strong potential in practical applications.

## 1. Introduction

Cables are key structural components of cable-stayed, suspension, and tied-arch bridges, which directly affect the safety and performance of the entire system. Accurately identifying the dynamic parameters and tension of stayed cables is essential in structural health monitoring (SHM) for these bridge structures, enabling early detection of damage and long-term condition assessment [1,2]. Existing traditional vibration-based methods rely on contact sensors to measure acceleration, which are costly and labor-intensive to install and maintain [3]. In recent years, the advent of low-cost, high-resolution imaging devices and advances in machine vision technology have made vision-based approaches increasingly viable for structural health monitoring (SHM) of cables [4,5]. However, there is currently no solution for comprehensively identifying the dynamic parameters and tension of stayed cables based on machine vision methods, which also highlights the strong engineering needs for efficient, non-contact approaches to support routine cable monitoring practices.

Vision-based techniques for structural monitoring and assessment have garnered increasing attention in both research and practice [6,7]. For instance, Xu et al. [8] utilized a template-matching approach based on zero-mean cross-correlation to track the dynamic displacement of a cable-stayed bridge under pedestrian loading, analyzing the instantaneous variations in frequency and amplitude during large-scale pedestrian movement. Zhao et al. [9] integrated the support-based filtering algorithm with the Lucas–Kanade optical flow to estimate the vibration response of a scaled model of a cable-stayed bridge tower under seismic loading. Similarly, Dong et al. [10] combined the Lucas–Kanade optical flow technique with SIFT key points to monitor the displacement of a stadium structure under jumping-induced loads during a football match. These technological developments have rendered machine vision approaches increasingly viable for SHM tasks, facilitating the extraction of meaningful information about structural conditions and offering valuable insights for routine monitoring practices [11,12].

Vision-based displacement measurements allow for the extraction of cable vibration frequencies or deformations, enabling the estimation of cable forces through the analysis of the frequency–tension relationship. In previous studies [13,14,15], vibration frequencies derived from vision-based displacement data were utilized to estimate cable tensions. Specifically, Kim et al. [14] employed a zero-mean normalized cross-correlation (ZNCC)-based template-matching method to monitor vibrations of multiple cables on a cable-stayed bridge, successfully estimating cable tensions from the measured frequencies. Feng et al. [13] applied frequency-domain template matching to track vibrations of stadium cable-stayed elements, from which cable forces are calculated. Zhao et al. [15] utilized a smartphone camera to track manually placed markers on the cables of a pedestrian bridge. By applying a sliding average filter to the displacement time series, vibration components induced by camera motion are suppressed, allowing accurate extraction of cable frequencies and subsequent estimation of cable tensions. However, current vision-based methods typically depend only on extracting the fundamental frequency of the cable, limiting the reliability of tension estimation due to the absence of multi-order modal verification.

Additionally, some studies have utilized multi-point displacement time series combined with operational modal analysis (OMA) techniques to extract structural modal parameters such as natural frequency, damping ratio, and mode shapes. For example, displacements at several structural points have been captured using a single camera, and modal parameters have been estimated based on the recorded displacement histories [16,17,18,19]. The mode shape of a cable-stayed bridge has been estimated by Ji and Chang [20] through the use of stereoscopic vision technology. Phase-based optical flow, Fourier transform, and motion magnification have been implemented by Chen et al. [21,22] to extract instantaneous mode shapes of beams in laboratory settings and truss bridges. Yang et al. [23,24] applied blind source separation, phase-based optical flow, motion magnification, and edge detection to analyze videos of small steel-frame structures and extract operational mode shapes. Additionally, motion magnification techniques were employed by Fioriti et al. [25] to extract mode shapes from several historic buildings. Tian et al. [26] employed machine vision-based displacement measurement methods to measure the displacement of beams during impact tests and extracted structural frequencies, scaling factors, and mode shapes. Hoskere et al. [27] employed drones to position cameras for measuring displacements at multiple local points across an entire bridge. Following a complete scan, the mode shapes of individual bridge segments are integrated to derive the global mode shape of the structure. Full-field displacement monitoring of wind turbine structures has been conducted using three-dimensional digital image correlation (3D-DIC) and multi-camera systems, from which operational deflection shapes are extracted [28,29,30]. Furthermore, Harmanci et al. [31] combined particle tracking velocimetry with phase-based motion magnification to capture the vibration response of shear frame structures subjected to shaker excitation, enabling the extraction of three-dimensional operational mode shapes.

Existing studies have demonstrated that vision-based structural dynamic monitoring offers significant advantages over traditional sensor-based approaches [11,32,33]. It circumvents the limitations imposed by the number and placement of sensors, reduces overall monitoring costs, and improves operational efficiency. Moreover, machine vision techniques enable the acquisition of dense and spatially continuous data, thereby enhancing the spatial resolution of vibration monitoring [5,6]. On one hand, stay cables are slender structural components, and the sparse tracking of isolated points fails to represent the continuous deformation along the entire length of the cable, making it insufficient for identifying holographic dynamic parameters. Moreover, reliable tension identification typically requires multiple modal frequencies for calculation and cross-verification to ensure stability and reduce the impact of potential systematic errors. However, current vision-based methods usually rely solely on the fundamental frequency, which limits the accuracy and robustness of the tension estimation. On the other hand, vibrations of stayed cables are typically very weak, especially for higher-order modes. While vision-based approaches have the potential to detect fine-scale motions, the weak amplitude of these signals poses difficulties for accurately capturing higher-order modal responses. Consequently, these approaches fall short in providing a comprehensive understanding of the full-field dynamic behavior and tension states of cables in real-world bridge structures [19,34].

In this study, a novel holographic vision-based method is proposed, which enables high-precision identification of the high-order full-field dynamic parameters of stayed cables, including the high-order natural frequencies, damping ratios, holographic modal shapes, dynamic displacement vectors, as well as cable tensions. The holographic vision sensor (HVS) is applied to acquire the spatiotemporal sequence data of the stayed cables, and to arrange dense and continuous pixel-level or subpixel-level virtual measurement feature points on the surface of the monitored structure [6,7]. The proposed full-field optical flow tracking algorithm is used to track the optical flow field changes of the continuous motion signal spectral components of holographic feature points. The proposed holographic feature point video magnification algorithm is applied to enhance weak visual motion signals of the stayed cables. This study aims to identify the high-order full-field dynamic parameters of stayed cables by the support of the proposed method, enabling high-precision estimation of cable forces.

The remainder of this paper is organized as follows. Section 2 provides an overview of the theoretical foundation for the proposed holographic vision-based method and the holographic vision sensor. Section 3 describes the overall experimental setup, and the workflow of the proposed holographic vision-based method is also introduced. In Section 4, we present experimental results that validate the effectiveness of the proposed approach in identifying the high-order full-field dynamic parameters and tension of the stayed cables. Finally, Section 5 summarizes the conclusions of this study and highlights its potential for comprehensive monitoring of the stayed cables.

## 2. Theoretical Background

The full-field dynamic parameters of the stayed cables primarily include natural frequencies, damping ratios, holographic modal shapes, and dynamic displacement vectors, which all characterize the dynamic behavior of the stayed cables [32]. Furthermore, by continuously tracking the full-field dynamic parameters under varying tension states, the cable force can be rapidly and accurately estimated based on the well-built mathematical relationship between modal parameters and cable force [33,34].

### 2.1. Full-Field Optical Flow Tracking Algorithm for the Motion Signal Components

Assuming a series of dense and continuous virtual-measurement holographic feature points *P* are obtained from the surface of the stayed cables, the continuous motion signal spectral components of these discrete holographic feature points can characterize changes in the physical properties of the structure. When the monitored cable undergoes vibration due to external excitations such as loads or environmental effects, the continuous motion signal spectral components of the holographic feature points will cause changes in the optical flow field over a time interval Δt in spatial and temporal scales. Thus, the frame difference algorithm [22,35] can be used for full-field optical flow tracking [36,37] and dynamic analysis.

**Figure 1:**
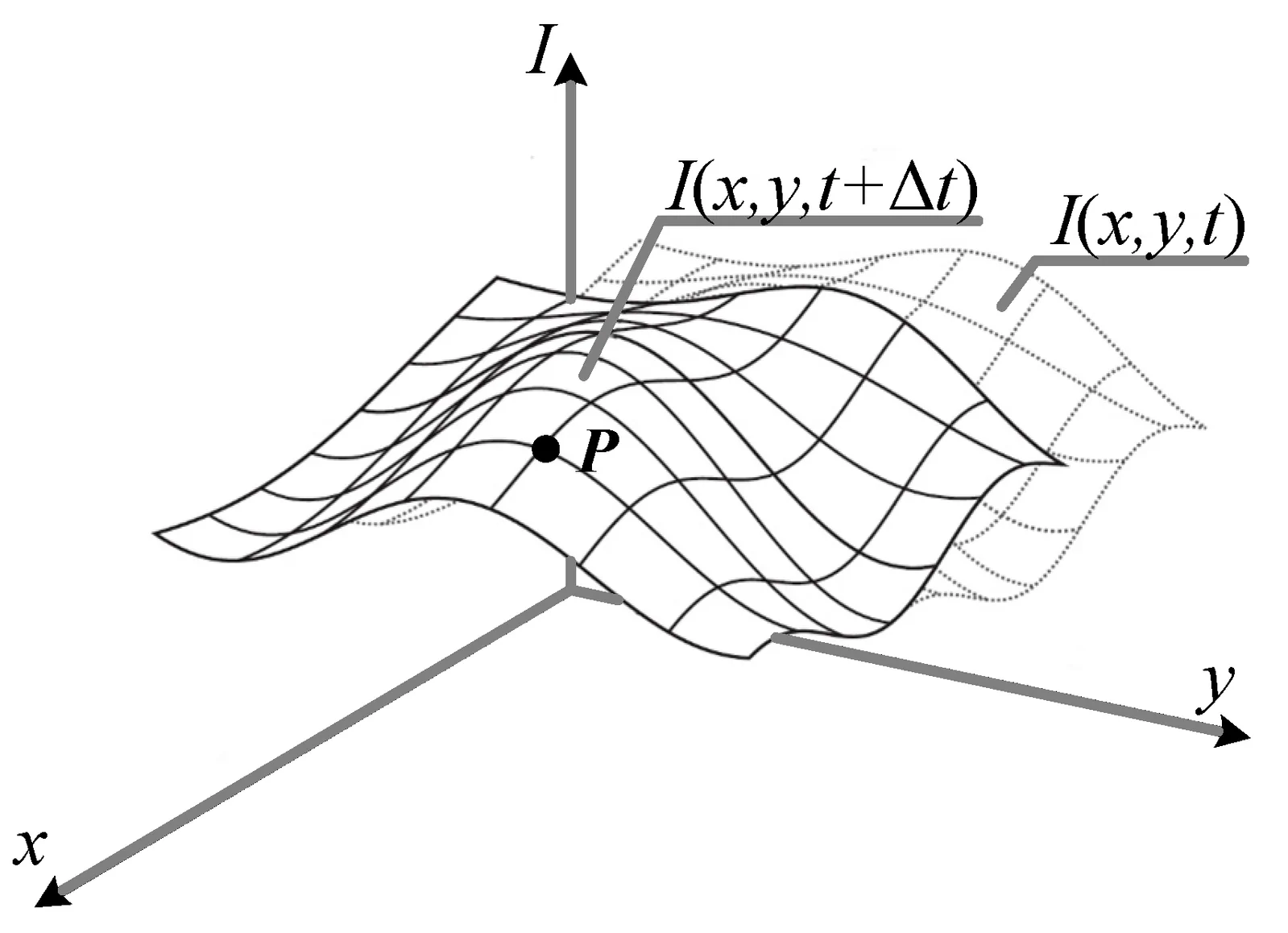
The optical flow field changes with time.

The full-field optical flow I(x,y,t) represents the characteristics of the monitored structure surface over time, assuming that the holographic feature points experience position changes of x+Δx and y+Δy within time Δt, while maintaining brightness constancy, as illustrated in Figure 1.

The constraint equation of the full-field optical flow is as follows:

(1) I(x,y,t)=I(x+Δx,y+Δy,t+Δt)

The above equation can be expanded into a Taylor series form:

(2) $$I(x+\Delta x,y+\Delta y,t+\Delta t)=I(x,y,t)+\frac{\partial I}{\partial x}\Delta x+\frac{\partial I}{\partial y}\Delta y+\frac{\partial I}{\partial t}\Delta t+\varepsilon$$

where ε is a high-order term. According to the conservation of optical flow brightness and the negligible deformation of structural vibration, the partial derivatives in the *x*, *y*, and *t* directions can be denoted as $f_{x}=\partial I/\partial x,f_{y}=\partial I/\partial y$ and $f_{t}=\partial I/\partial t$ in the spatial domain, respectively. The full-field optical flow vector is Δx=dx/dt and Δy=dy/dt, and Equation (2) can be written as follows:

(3) $$f_{x}\Delta x+f_{y}\Delta y+f_{t}\Delta t=\frac{\partial I}{\partial x}\Delta x+\frac{\partial I}{\partial y}\Delta y+\frac{\partial I}{\partial t}\Delta t=0$$

For the discrete problems, Δt•∂I/∂t is the function of illumination intensity change between pixel elements in a single frame of the spatial–temporal sequence data according to the frame difference algorithm [32,36].

(4) $$\frac{\partial I}{\partial t}\Delta t=I(x,y,t+\Delta t)-I(x,y,t)$$

Substituting Equation (4) into Equation (3) gives

(5) $$\frac{\partial I}{\partial x}\Delta x+\frac{\partial I}{\partial y}\Delta y=-\frac{\partial I}{\partial t}\Delta t=I(x,y,t)-I(x,y,t+\Delta t)$$

**Figure 2:**
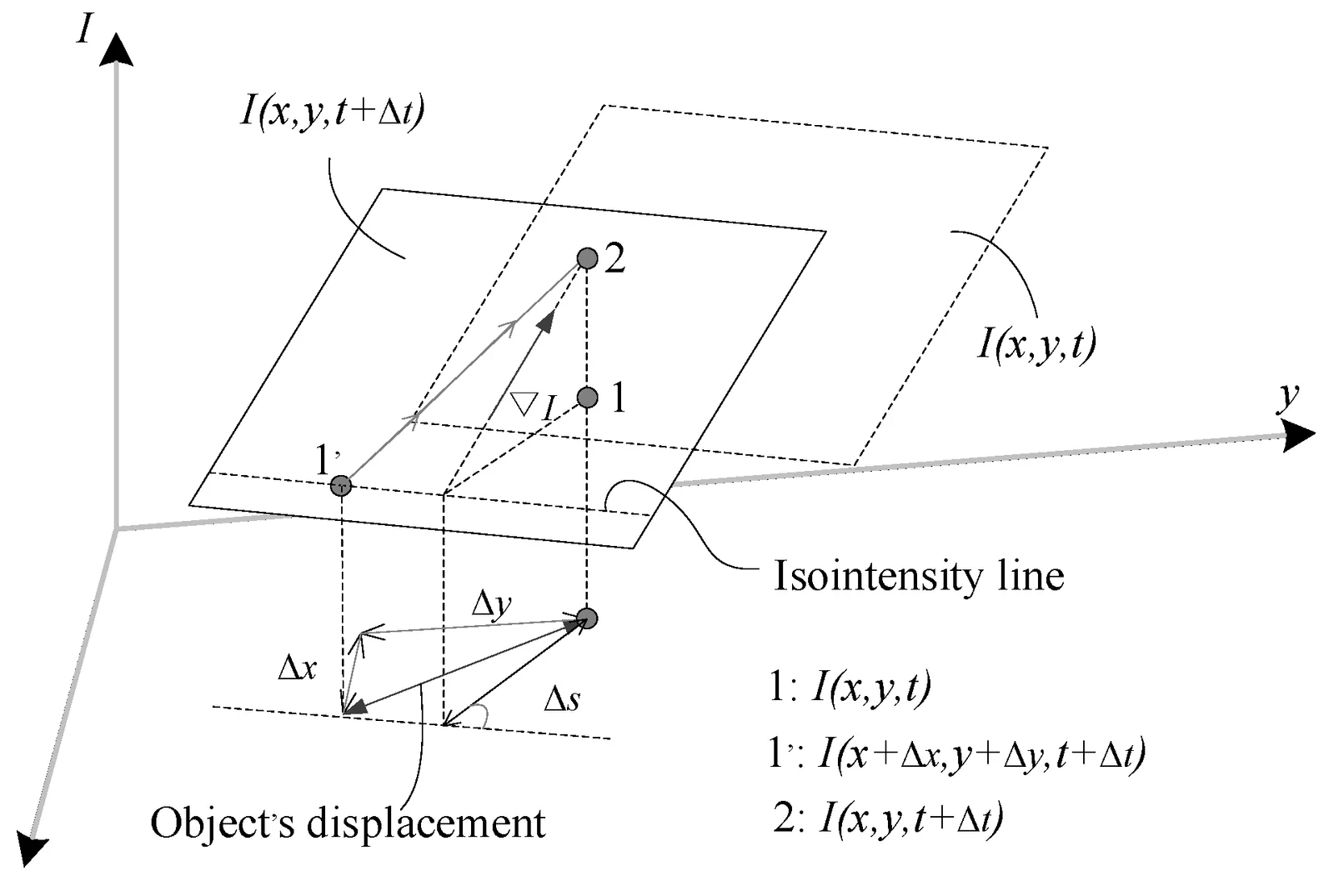
The displacement of the optical flow field and the change in illumination intensity of the selected pixel element $\left(x_{j},y_{k}\right)$.

In Equation (5), the unknown parameters *f_x_*, *f_y_*, and *f_t_* can be solved from the known pixel elements of a single frame, but the unknown full-field optical flow vector Δx and Δy cannot be directly solved by the full-field optical flow constraint equation. The gradient change information of adjacent pixel elements is introduced, which provides the displacement information in the *x* and *y* directions. So, the second constraint equation can be used to limit the observation to the displacement in the direction of the illumination intensity gradient, which is denoted as below [22,32,35,36,37].

(6) $$\left|\nabla I\right|\Delta s=I(x,y,t)-I(x,y,t+\Delta t)$$

(7) $$\left|\nabla I\right|\Delta s=\sqrt{{\left(\frac{\partial I}{\partial x}\right)}^{2}+{\left(\frac{\partial I}{\partial y}\right)}^{2}}$$

where the $\left|\nabla I\right|$ is a known scalar value of the gray component gradient, which can be calculated from the partial derivatives of the first sequence image, *∆s* is an unknown scalar value of the displacement in the direction of the illumination intensity gradient, as shown in Figure 2.

By observing the pixel element’s optical flow intensity between the initial sequence image $I_{0}(x,y)$ and the subsequent sequence image I(x,y,t), as well as the linear relationship between the displacements and the optical flow intensity, the displacements of the initial sequence image can be calculated.

(8) $$s(x,y,t)=\frac{\left|I_{0}(x,y)-I(x,y,t)\right|}{\left|\nabla I_{0}\right|}$$

(9) $$s(x,y,t)=\frac{\left|I_{0}(x,y)-I(x+\Delta x_{L},y+\Delta y_{L},t)\right|}{\left|\nabla I_{0}\right|}+\sqrt{{\left(\Delta x_{L}\right)}^{2}+{\left(\Delta y_{L}\right)}^{2}}$$

Then, the full-field optical flow vectors $\Delta x_{L}$ and $\Delta y_{L}$ can be obtained as below, and *round* (·) is the rounding function:

(10) $$\Delta x_{L}=round\left(\frac{\partial I}{\partial x}•\frac{1}{\left|\nabla I_{0}\right|}\right),\;\Delta y_{L}=round\left(\frac{\partial I}{\partial y}•\frac{1}{\left|\nabla I_{0}\right|}\right)$$

According to Equation (10), the full-field optical flow vector can be used to track the displacement information of the holographic feature points of the measured structure in both time and space. The displacement information can be converted into pixel displacement through the continuous motion signal spectral components. Finally, the pixel displacement is converted into the actual displacement in the measurement coordinate system. This is an important theoretical basis for identifying the high-order full-field dynamic parameters of the stayed cables.

### 2.2. Weak Visual Motion Signal Amplification Algorithm

Theoretically, the full-field dynamic parameters of the stayed cables can be extracted from the dynamic response, which is obtained by analyzing the motion signal spectral components of the holographic feature points. However, in large-scale structures like cable-stayed bridges, vibrations caused by low-energy excitation are often weak. Consequently, these weak responses are likely to be missed or misrepresented during data processing, which can lead to inaccuracies in identifying higher-order modes, thereby affecting the visualization and extraction of the holographic modal shapes and dynamic displacement vectors of the stayed cables. Therefore, this paper proposes a holographic feature point video magnification algorithm (HFPVM) to enhance the weak visual motion signals to visualize the morphological motion information of the stayed cables.

**Figure 3:**
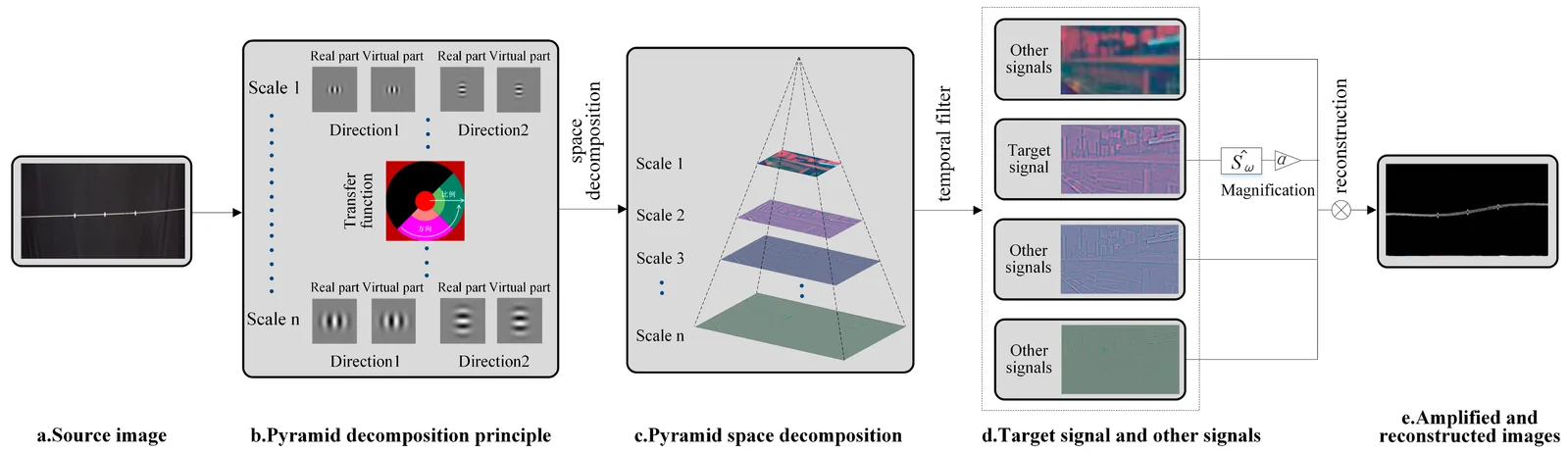
Motion signal component of the holographic feature point amplification based on HFPVM.

The principle of HFPVM is to leverage the assumption of spatial consistency of full-field optical flow and brightness constancy in optical flow intensity. In this method, the weak structural responses are equivalent to the subtle changes in optical flow intensity and spatial position of holographic feature points. By directly analyzing the motion mode and different signal compositions of the full-field optical flow, the specific morphological motion information of the stayed cable can be amplified and extracted. Specifically, the frequency-domain information is utilized to filter the spatial–temporal variation characteristics of motion signals, which allows for the amplification of the morphological motion information corresponding to specific frequency bands. This process facilitates the visualization and extraction of structural holographic modal shapes and dynamic displacement vectors. The principle of HFPVM is shown in Figure 3.

Taking a one-dimensional signal as an example, the signal intensity of a pixel at any point *x* in the holographic feature point set *P* of the stayed cables at time *t* can be expressed as $I_{p}(x,t)$.

(11) $$I_{p}(x,t)=f(x-\delta (t))\;t>0,\;I_{p}(x,0)=f(x)\;t=0$$

where the *δ*(*t*) represents the displacement function of a small amplitude generated by the weak structural response. The weak structural response in $I_{p}(x,t)$ is separated and expanded in the form of the first-order Taylor series, and this is superimposed with the original input signal $I_{p}(x,t)$ after amplifying a specific motion signal component. At time *t*, the first-order Taylor series form of $I_{p}(x,t)$ with respect to *x* can be expanded as follows:

(12) $$I_{p}(x,t)=f(x-\delta (t))\approx f(x)-\frac{\partial f(x)}{\partial x}\delta (t)$$

(13) $$B_{p}(x,t)=-\frac{\partial f(x)}{\partial x}\delta (t)$$

Then, the output signal after amplifying $B_{p}(x,t)$ by α times is $I_{p}^{\prime }(x,t)$.

(14) $$I_{p}^{\prime }(x,t)=I_{p}(x,t)+\alpha B_{p}(x,t)=I_{p}(x,t)-\alpha \frac{\partial f(x)}{\partial x}\delta (t)$$

Its first-order Taylor series approximation is as follows.

(15) $$I_{p}^{\prime }(x,t)=f(x-(1+\alpha )\delta (t))=I_{p}(x,t)+\alpha B_{p}(x,t)\approx f(x)-\frac{\partial f(x)}{\partial x}\delta (t)-\alpha \frac{\partial f(x)}{\partial x}\delta (t)$$

**Figure 4:**
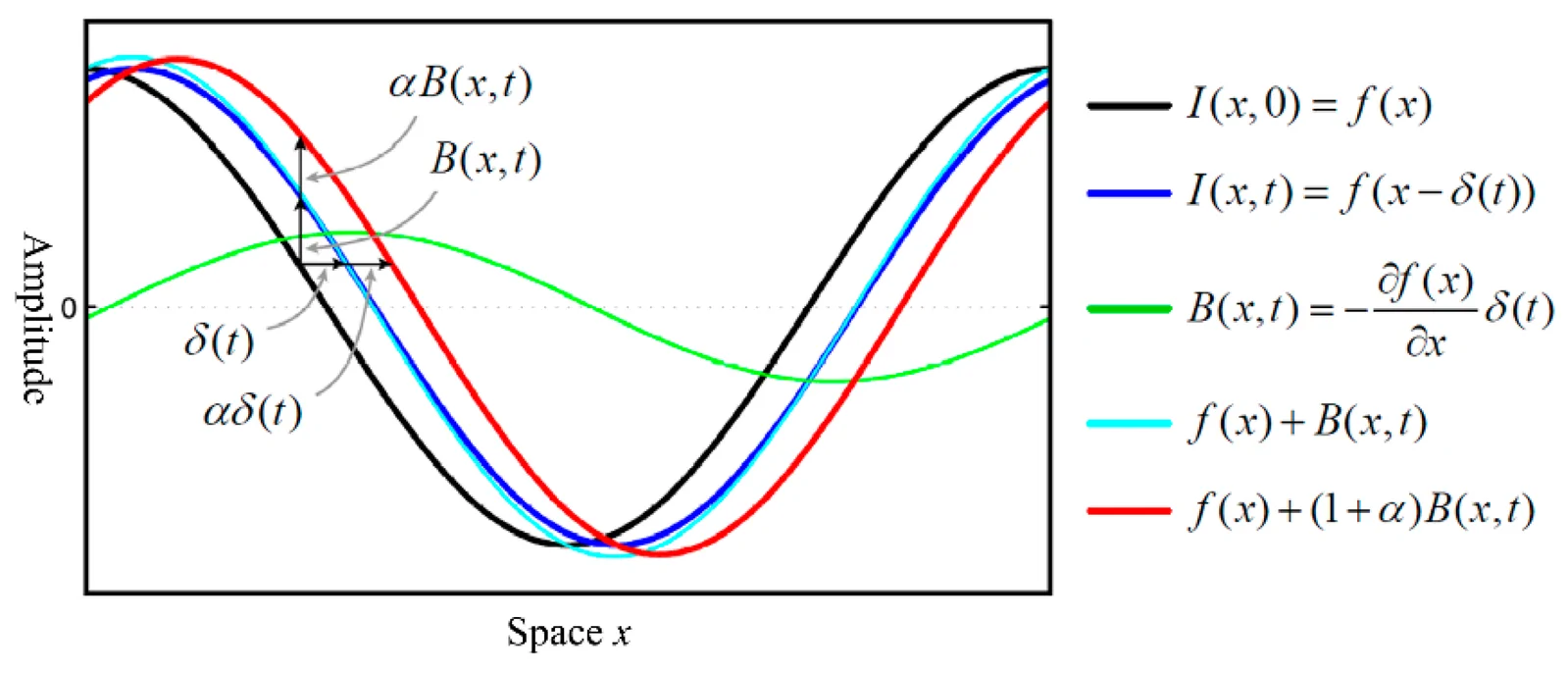
The calculation results of the one-dimensional sine signal through amplification.

The calculation results of the one-dimensional sine signal through amplification are shown in Figure 4.

Using frequency-domain bandpass filters [38,39] to amplify the motion signal components of holographic feature points within a specific frequency band, Equation (11) is converted to the frequency domain. Since only the motion signal components of holographic feature points are tracked and amplified, the motion can be considered global. Therefore, the global Fourier transform [39] can be directly applied to decompose the digital image sequence data into sinusoidal signals. For amplification of local motion, a controllable pyramid [38,39] is used to decompose digital image sequence data into a series of base function signals at different spatial scales and in different directions, as shown in Figure 3.

(16) $$f(x)=\sum_{\omega }A_{\omega }e^{i\phi_{\omega }}e^{-i\omega x}$$

where ω and $\phi_{\omega }$ are the frequency and phase corresponding to the decoupled motion signal components, respectively. According to the translational property of the FFT,

(17) $$I_{p}(x,t)=f(x-\delta (t))=\sum_{\omega }A_{\omega }e^{i\phi_{\omega }}e^{-i\omega (x-\delta (t))}=\sum_{\omega }A_{\omega }e^{i(\phi_{\omega }+\omega \delta (t))}e^{-i\omega x}$$

For the frequency ω corresponding to the motion signal component, the complex exponential form $S_{\omega }(x,t)$ can be expressed as follows:

(18) $$S_{\omega }(x,t)=\sum_{\omega }A_{\omega }e^{i(\phi_{\omega }+\omega \delta (t))}e^{-i\omega x}$$

When *t* = 0, the corresponding phase is $\phi_{\omega }-\omega \delta (t)$, and the phase difference at time *t* is as follows:

(19) $$B_{\omega }(x,t)=\omega \delta (t)$$

In a specific frequency band, the weak motion signal component of the holographic feature point $B_{\omega }(x,t)$ is amplified by α times:

(20) $$B_{\omega }^{\prime }(x,t)=\alpha \omega \delta (t)$$

Then, the output signal after amplification of the holographic feature point motion signal component corresponding to the specific frequency band after decoupling in the frequency domain is $I_{p}^{\prime }(x,t)$ multiplied by α:

(21) $$I_{p}^{\prime }(x,t)=f(x-(1+\alpha )\delta (t))=I_{p}(x,t)+\alpha B_{p}(x,t)=\sum_{\omega }A_{\omega }e^{i(\phi_{\omega }-(x-(1+\alpha )\omega \delta (t)))}$$

To select an appropriate amplification factor α, a discriminative formula was proposed in study [40] to guide the amplification of the visual motion information, giving the largest motion amplification factor, α, compatible with accurate motion magnification of a given video motion *δ*(*t*) and image structure spatial wavelength λ, where the λ=2π/ω; the discriminative formula is given below:

(22) $$\left(1+\alpha \right)\delta \left(t\right)<\frac{\lambda }{8}$$

In our study, the research conclusion of Wu et al. [40] is adopted, and the amplification factor is set according to the maximum compatible gain based on different center frequencies, that is, adaptively selecting the amplification factor with the equation $\left(1+\alpha \right)\delta \left(t\right)=\lambda /8$.

**Figure 5:**
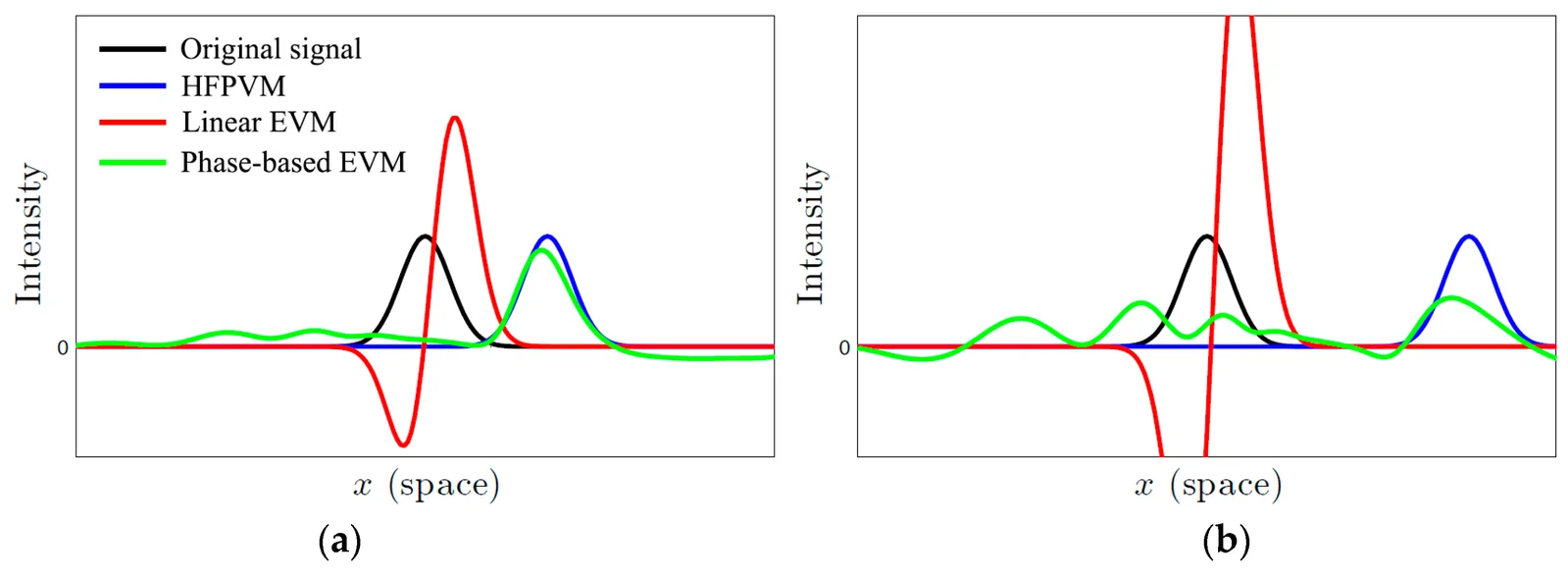
Comparison of the one-dimensional sine signal through amplification. (**a**) α = 6. (**b**) α = 14.

The calculation results of a one-dimensional sinusoidal signal based on HFPVM and other traditional algorithms under different amplification factors are shown in Figure 5.

As shown in Figure 5, linear EVM not only enhances the spatial position of the motion signal but also amplifies the optical flow intensity. However, as the amplification factor increases, the enhancement of motion information remains limited, while the optical flow intensity may exceed acceptable bounds. In contrast, PVM effectively amplifies motion signals, but it should be noted that excessive amplification factors can lead to distortions or artifacts in the final results. The proposed HFPVM combines holographic feature point tracking and the frequency-domain filtering mechanism, allowing it to amplify signals within specific frequency bands. As the amplification factor increases, the motion signal continues to be enhanced without introducing significant anomalies. Moreover, HFPVM largely maintains the stability of optical flow intensity, indicating that it does not introduce noticeable image distortion during the image signal reconstruction. It is worth noting that HFPVM is an improved version of the phase-based Eulerian video magnification method. However, when the original motion signal is weak or the amplification factor is not appropriately selected, there remains a risk of distortion in the amplified results.

Compared to conventional linear Eulerian video magnification (linear EVM) and phase-based Eulerian video magnification (PVM) algorithms [41,42,43,44], only the subtle variations in optical flow intensity and spatial position of the holographic feature points are handled with the proposed HFPVM. After decoupling in the time and frequency domains, it allows for the selective amplification of weak structural responses while avoiding the amplification of motion data from subsidiary structures or environmental signals. This selective method effectively reduces noise from irrelevant motion, significantly enhancing computational efficiency and demonstrating strong robustness as well as resistance to noise and disturbance. Therefore, it is especially effective in amplifying slight responses of primary structural components in cable-supported bridges, where the testing environment is relatively straightforward.

### 2.3. Holographic Visual Sensor

**Figure 6:**
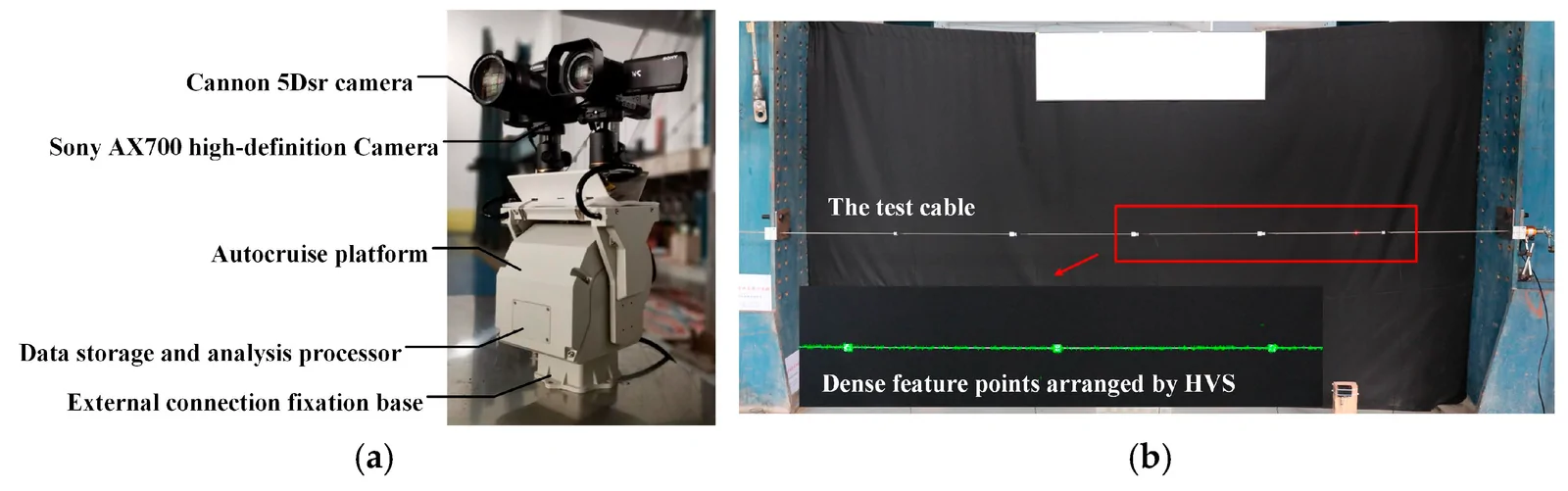
Proposed holographic visual sensor (HVS): (**a**) main components of the sensor and (**b**) the holography feature points used for tracking.

The holographic visual sensor (HVS), proposed in previous studies [6,32], enables high-resolution, non-contact monitoring, which has been proven advantageous for vision-based structural health monitoring, particularly in enhancing data visualization and real-time analysis. In this study, the HVS is used as a tool for obtaining the spatial–temporal sequence data of the stayed cable. In addition, the dense holographic feature points and continuous motion signal spectral components can also be defined by this equipment across the surface of the monitored stayed cables. The HVS is composed of a Canon 5Dsr camera and a Sony AX700 high-definition camera, as shown in Figure 6a. The motion signal spectral component of the holographic feature points for target tracking is shown in Figure 6b. Details of the data preprocessing procedures can be found in previous studies [6,32], which provide the foundation for the proposed framework in visual tracking and amplification of weak cable vibrations, aimed at identification of full-field dynamic parameters and tension estimation.

It is worth noting that this study focuses on identifying the full-field dynamic parameters of the stayed cables. Therefore, the Sony AX700 high-definition camera is employed to capture the dynamic response visual data, while the Canon 5DSr camera is not utilized in this study. Before holographic testing, both internal and external camera parameters are calibrated using a standard chessboard target with alternating black and white squares. The calibration results of the HVS parameters are presented in Table 1.

## 3. Experimental Study

### 3.1. Description of the Laboratory Setup

**Figure 7:**
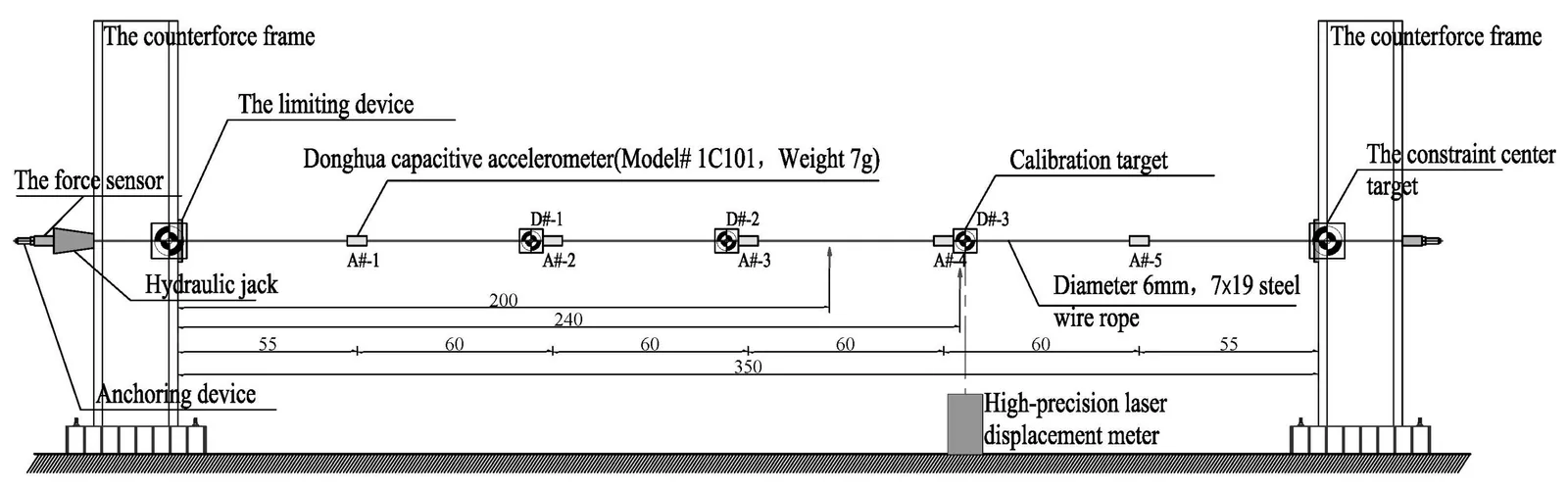
The schematic diagram of the arrangement and testing of various sensors.

**Figure 8:**
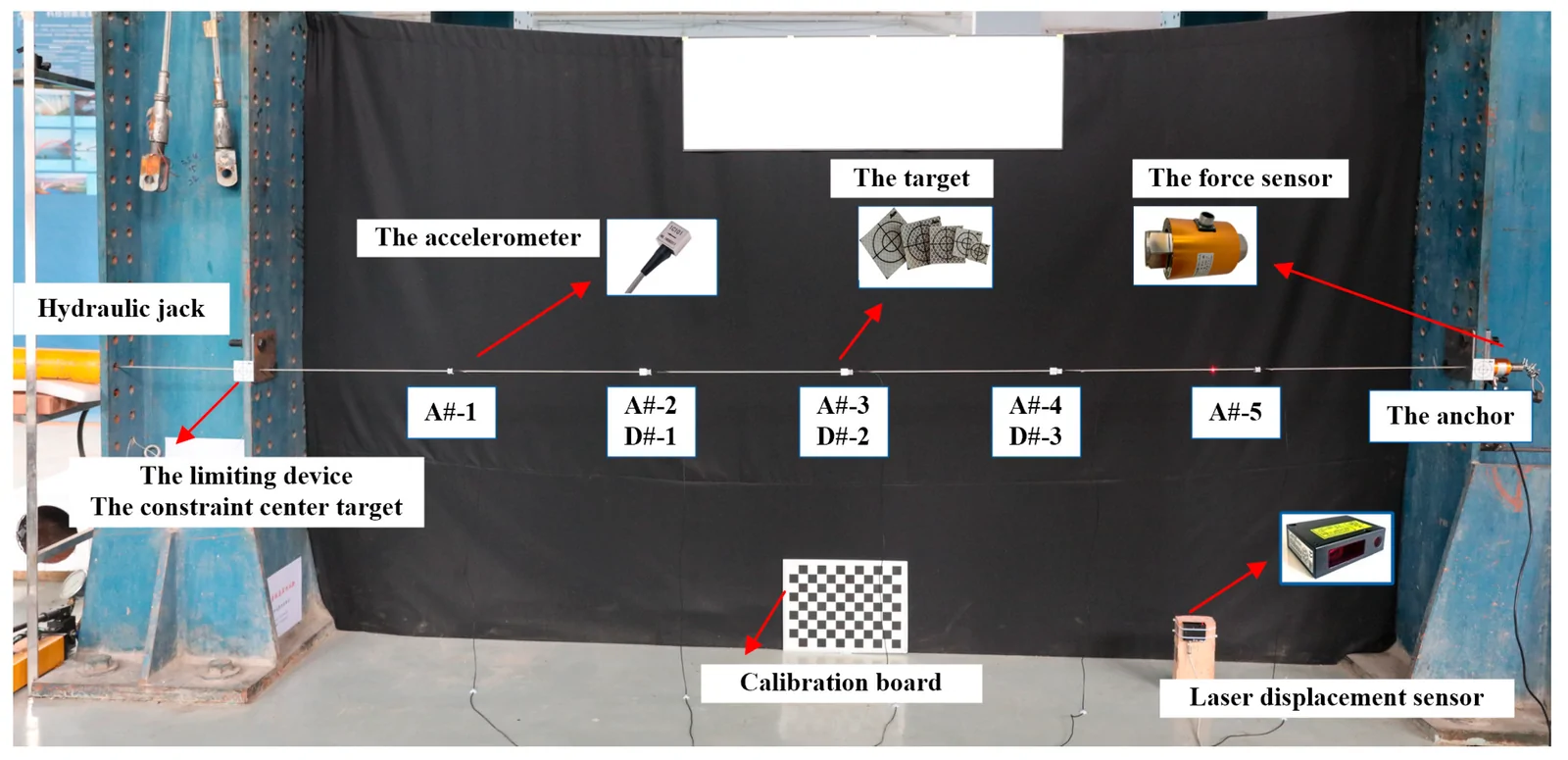
The layout of the test site.

A series of experiments were conducted on the stayed cable models. A stainless-steel wire rope with a diameter of 6 mm and a 7 × 19 strand configuration was used as the scaled model, with the corresponding parameters listed in Table 2. The schematic diagram of sensor arrangement and instrumentation setup is shown in Figure 7, while the overall layout of the experimental site is presented in Figure 8.

The test conditions are specified in Table 3. Due to the influence of excitation types and stochastic factors in data processing (including sampling, recording, and analysis), the holographic characteristics of the stayed cable are represented by the average values obtained from multiple repeated tests. To ensure reliability, the deviation of any single test result from the average must remain within a specified tolerance range. In this study, the acceptable deviation is set at ±3%.

### 3.2. Workflow for the Full-Field Dynamic Parameter Identification and Tension Estimation

Based on the full-field optical flow tracking algorithm, the displacement information of the continuous motion signal spectral components of the holographic feature points in both time and space is obtained. These dynamic displacement signals are then used for frequency-domain analysis to capture the natural frequencies and damping ratios. During the analysis, each point in the spatial point set is continuously accessed to compute the corresponding local geometric displacements.

By establishing the relationship between the vibration frequency and the tension of the stayed cable, the cable force can be estimated by using the vibration frequency of the cable. The frequency method [45] for cable force estimation is given as Equation (23):

(23) $$T=\frac{4\rho L^{2}f_{n}^{2}}{n^{2}}-\frac{n^{2}\pi EI}{L^{2}}(n=1,2,3,\ldots )$$

where *f*_n_ is the *n*-order natural frequency of the cable, *L* is the calculated length of the cable string, *n* is the order of the natural frequency, *EI* is the bending stiffness of the cable, and ρ is the line density. In engineering applications, the natural frequency *f*_n_ needs to be solved according to the full-field dynamic parameters. It is worth noting that the general calculation formula of the frequency method is considered in this paper, and the modification of this formula is not considered for the moment when considering the cable sag effect, damping, and complex boundary conditions. However, it is necessary to select an appropriate cable force calculation model for actual bridge structures based on the actual boundary conditions to ensure the accuracy of the estimated cable force.

To further visualize the morphological motion information of the stayed cables, i.e., the holographic mode shapes and dynamic displacement vectors, the proposed HFPVM method is employed to amplify the weak motion response embedded within digital image sequences. This approach significantly enhances the visual perception capability. Specifically, by decoupling the signals in both the time and frequency domains and considering the inherent dynamic characteristics of the stayed cable, suitable bandpass filters and amplification coefficients can be designed. This not only effectively suppresses irrelevant signals outside the passband but also amplifies the weak vibration responses associated with the structural dynamics, thereby improving their observability.

**Figure 9:**
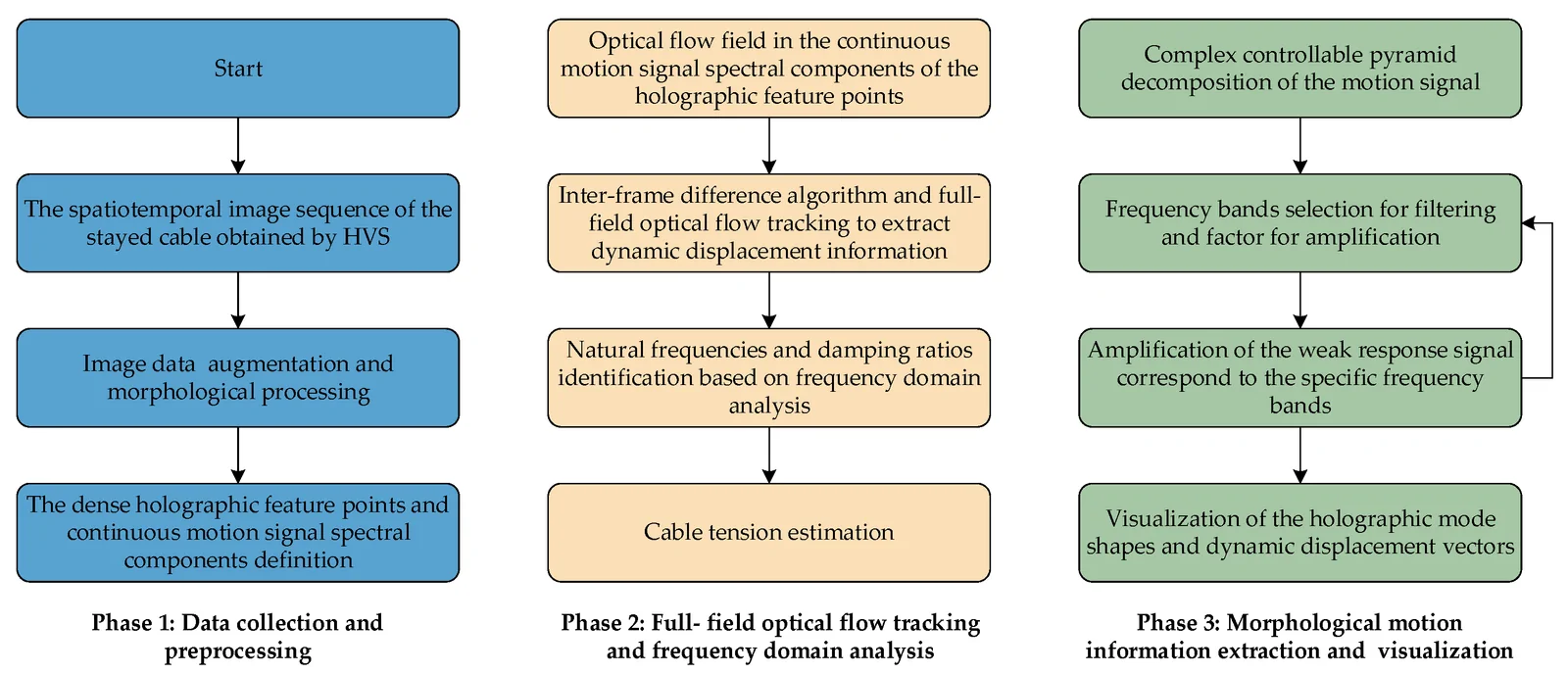
Workflow for full-field dynamic parameter identification and cable force estimation.

Additionally, the holographic feature points are continuously tracked and analyzed for their local mechanical responses. The selected frequency bands, phase information, and amplified amplitudes can be integrated with the dynamic displacement to reconstruct the dynamic displacement vector of the stayed cable. By integrating the methods introduced in Section 2.1 and Section 2.2, a comprehensive monitoring workflow for the stayed cables via full-field dynamic parameters identification and tension estimation is established shown in Figure 9.

The detailed workflow is illustrated as follows:

Step 1: The sequence of spatial–temporal images of the stayed cables is captured using the HVS.

Step 2: The HVS system’s image preprocessing functions are utilized to address image degradation issues, such as blurred or discontinuous edge contours, by implementing data augmentation and morphological processing.

Step 3: The dense holographic feature points and continuous motion signal spectral components on the surface of the monitored cables are defined using the processed image data. The full-field optical flow tracking algorithm is employed, as mentioned in Section 2.1, to trace these continuous holographic feature points.

Step 4: The changes in the optical flow field for the continuous motion signal spectral components of the holographic feature points are analyzed with the time history. The inter-frame difference algorithm and full-field optical flow tracking algorithm are used to extract dynamic displacement information. Then, the frequency-domain analysis and the covariance-driven stochastic subspace identification method (Cov-SSI) [46] are used to capture the natural frequencies and damping ratios, so that the cable tension can be accurately estimated.

Step 5: The single-frame image is decomposed into a series of basic function signals at different spatial and direction scales by the complex controllable pyramid decomposition.

Step 6: Based on the identified natural frequencies, a bandpass filter is designed to handle the decoupled motion signal. Specifically, the central frequency of the bandpass filter is aligned with the target mode’s identified frequency, and the bandwidth is set as ±0.2 Hz around the central frequency. Thus, the irrelevant signals outside the passband are suppressed, and the weak vibration responses associated with the structural dynamics are amplified.

Step 7: The filtering and amplification processes are iteratively performed until the weak structural response signals are enhanced to a predefined level. The amplified signals are then recombined with the original sequence to visualize the morphological motion information of the stayed cables, i.e., the holographic mode shapes and dynamic displacement vectors.

## 4. Results and Discussion

### 4.1. The Full-Field Dynamic Displacement and Frequency-Domain Analysis

**Figure 10:**
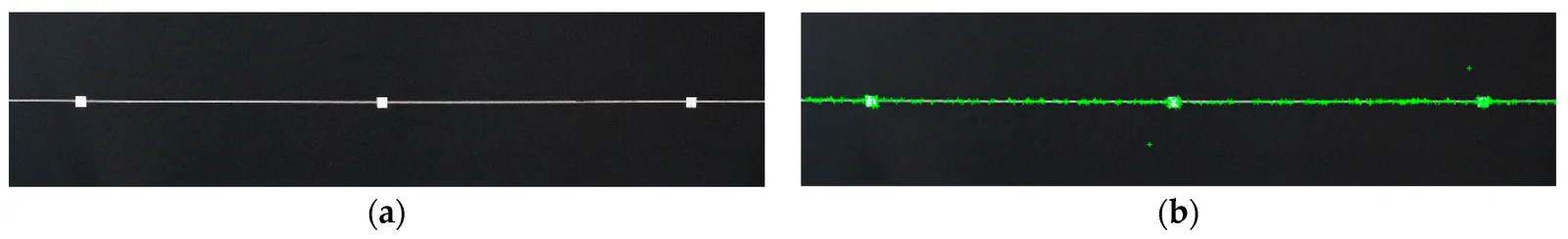
The continuous motion signal spectral components and the holographic feature points of the stayed cable: (**a**) continuous motion signal spectral components and (**b**) distributions of the holographic feature points.

**Figure 11:**
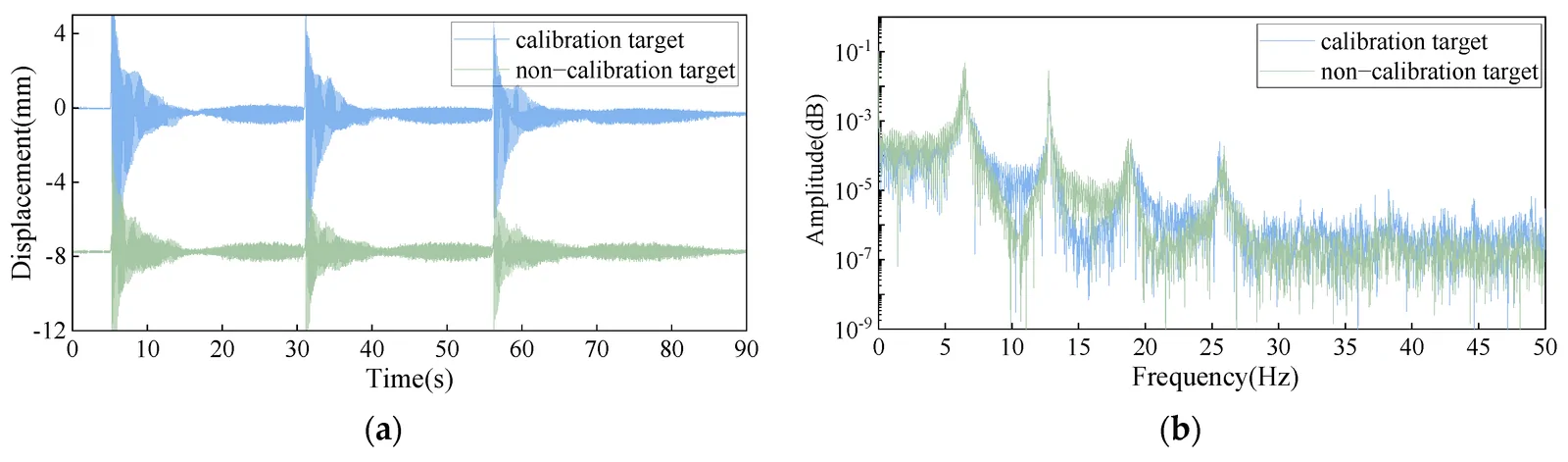

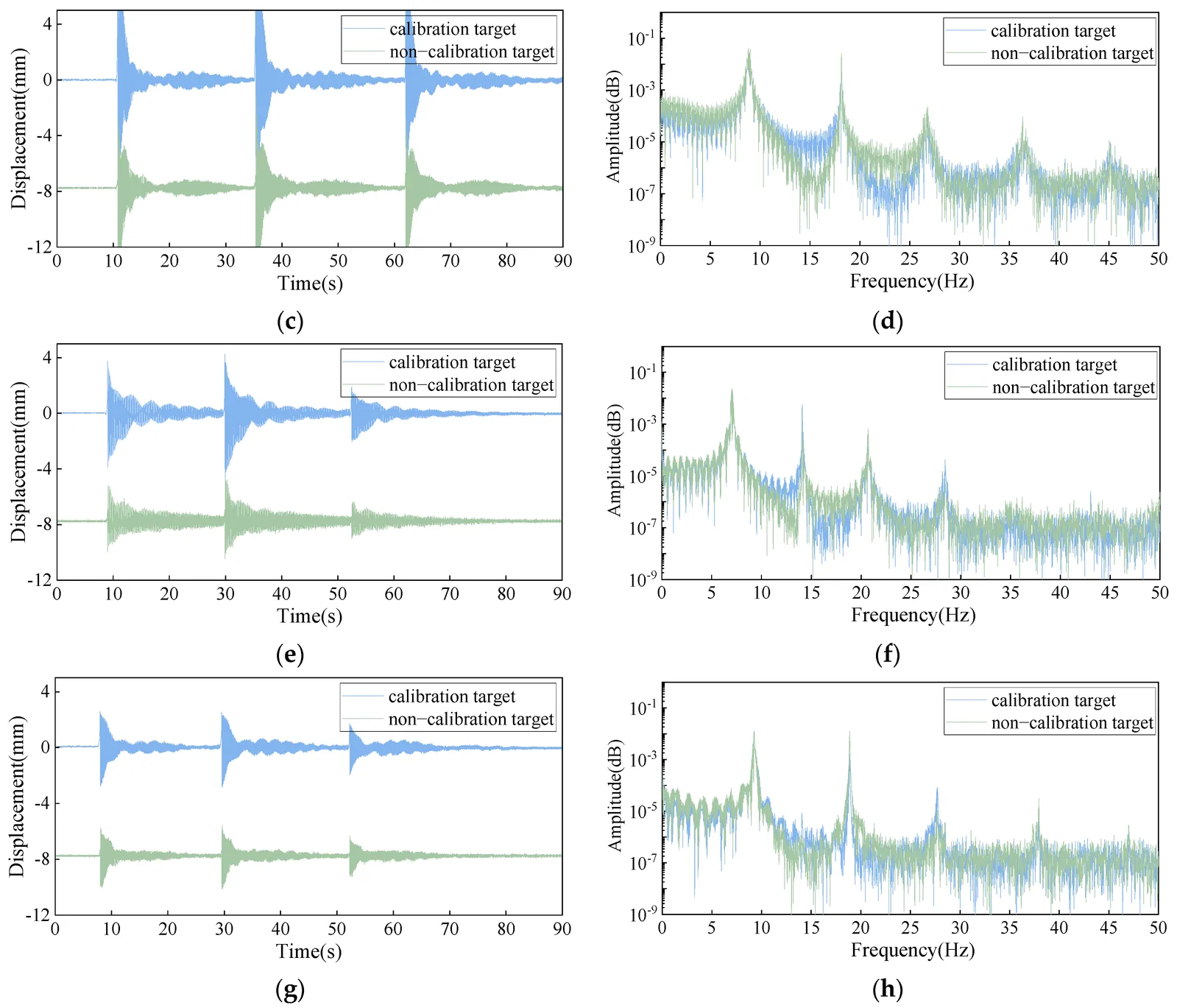
The test results at the D#-2 specific calibration target and the additional targets. (**a**) Structural response signal of A1 condition. (**b**) Spectral analysis result of A1 condition. (**c**) Structural response signal of A2 condition. (**d**) Spectral analysis result of A2 condition. (**e**) Structural response signal of B1 condition. (**f**) Spectral analysis result of B1 condition. (**g**) Structural response signal of B2 condition. (**h**) Spectral analysis result of B2 condition.

The HVS is employed to capture the dynamic response visual data of the stayed cable, and the sample rate is 100 Hz. The continuous motion signal spectral components and the holographic feature points of the stayed cable are presented in Figure 10. To evaluate the effectiveness of the proposed algorithms, the multi-target calibration points D#-1, D#-2, and D#-3 are used to extract the corresponding dynamic displacement. Subsequently, frequency-domain analysis is conducted on these signals. In this experiment, the impact of changes in lighting conditions on optical flow tracking has not been considered yet, as the laboratory environment’s lighting conditions remained relatively stable over short durations. The frequency-domain analysis results for the D#-2 and the non-calibration target are presented in Figure 11.

As illustrated in Figure 11, the dynamic displacement signals for both calibrated and non-calibrated targets exhibit similar oscillation patterns under various conditions. However, it can be observed that there is a deviation between the displacement of the calibrated and non-calibrated targets, which is primarily due to the inconsistencies of their calibration standards. Overall, the displacement values extracted by the full-field optical flow tracking algorithm from holographic feature points are close to those extracted from calibration target points. Additionally, spectral analysis is conducted to reveal the frequency-domain characteristics of the dynamic displacements for both types of targets, as shown on the right side of Figure 11. The results show a consistent frequency domain, with both targets having the same peak frequencies. This demonstrates that the proposed full-field optical flow tracking algorithm can effectively capture the motion information of holographic feature points, accurately reflecting the local dynamic behavior of the stayed cable. Additionally, it is worth noting that the HVS can reliably utilize the surface texture of the stayed cable to arrange densely distributed holographic feature points for full-field displacement measurement, without the need for dedicated physical markers.

The natural frequencies of the stayed cables are obtained by the full-field optical flow tracking algorithm and frequency-domain analysis mentioned in Section 3.2, and the results of each experimental condition are shown in Table 4. The natural frequency and damping ratio results are the average value based on 60 independent tests, with non-calibrated targets at the corresponding locations of D#-1, D#-2, and D#-3. The time lag parameter and model order of the Cov-SSI algorithm are 5 and 50, respectively [46]. Under different test conditions, the signal-to-noise ratio and modal energy content of the dynamic signals may vary across different orders [46,47]. As a result, certain modes may fail to produce stable poles in the stabilization diagram when using Cov-SSI, making it difficult to extract reliable frequency and damping values for those modes [46,47]. For example, although the spectra shown in Figure 11b,d appear visually similar, the dynamic displacement signals under these two conditions exhibit considerable differences. This discrepancy may lead to difficulties in accurately extracting the 4th-order damping ratio under condition A1 using the Cov-SSI method, whereas a stable estimation was achieved under condition A2. Additionally, as shown in Table 4, differences are also observed between the results obtained using the holographic vision-based method and those from the accelerometer-based method. Notably, the accelerometer-based approach provides a stable estimation of the 4th-order damping ratio across all test conditions, while the holographic vision-based method sometimes fails to capture high-order damping ratios accurately.

Under the obtained results, the holographic vision-based method demonstrates excellent performance in identifying natural frequencies, exhibiting a relatively small error range. For instance, under the A1 condition, the average error in natural frequency is below 3%, with a maximum error of 2.2%, indicating the high accuracy of the holographic vision-based method in frequency identification. Compared to accelerometer results, the holographic vision-based method generally yields smaller errors across multiple conditions, particularly for higher-order modes, where greater stability is maintained. Holographic vision-based method also performs well in identifying damping ratios, with the average errors typically remaining below 20% across various test conditions. In specific cases, such as under the A2 and B2 conditions, the proposed method enables a more accurate estimation of both natural frequencies and damping ratios, reflecting the high precision achieved by this approach.

The analysis of the results indicates that the holographic vision-based method achieves high accuracy and stability in identifying natural frequencies and damping ratios. In particular, for higher-order modes, the errors remain small and consistent with both accelerometer data and theoretical values. The holographic vision-based method proves to be an effective and reliable tool for monitoring the stayed cable health, accurately capturing the frequency and damping characteristics. The findings demonstrate that, under a variety of test conditions, the displacement response trends and spectral analysis results for both calibrated and non-calibrated targets exhibit a high degree of consistency. The maximum average error in natural frequencies observed across multiple tests remains below 5%, thereby meeting the accuracy and stability requirements for practical engineering applications. The proposed method is shown to be capable of tracking displacement responses, estimating natural frequencies and damping ratios, and performing spectral analysis of the stayed cable motion signals at arbitrary positions.

### 4.2. Cable Tension Identification by Vibration Frequency Method

The natural frequencies of the stayed cables are successfully identified as shown in Section 4.1. Furthermore, the theoretical relationship between the cable tension and natural frequencies can be used to estimate the corresponding cable tension, enabling effective monitoring of the operational condition of the stayed cables. Based on Equation (23), the cable tension can be effectively calculated with the identified natural frequencies. Considering the influence of the additional sensor mass on the estimation of the stayed cable tension, we further revised the calculation formula by incorporating the total mass of the sensors and converting it into an equivalent increase in linear density based on the cable length. For comparison, the cable tension results measured by the pressure sensor are also listed in Table 5.

As shown in Table 5, the estimated cable tensions closely match the values measured by the pressure sensors under all four test conditions. The largest average relative error occurred under test condition B2, with the value of 3.76%. The maximum deviation in tension estimation is observed on the first-order frequency under test condition A1, reaching 6.67%. In addition, as shown in Table 5, some discrepancies exist between the measured and preset tension values due to the difficulty in precisely controlling the applied tension during testing. This suggests that certain minor systematic errors may have been introduced during the experimental process. Therefore, part of the discrepancy between the estimated and measured tension is due to the inaccuracy of the reference value itself. It is noted that such systematic errors can be reduced by using more precise instruments. As a result, the proposed holographic vision-based method can enable high-precision identification of cable tension, with an overall error maintained within 5%, thereby meeting practical engineering requirements.

**Figure 12:**
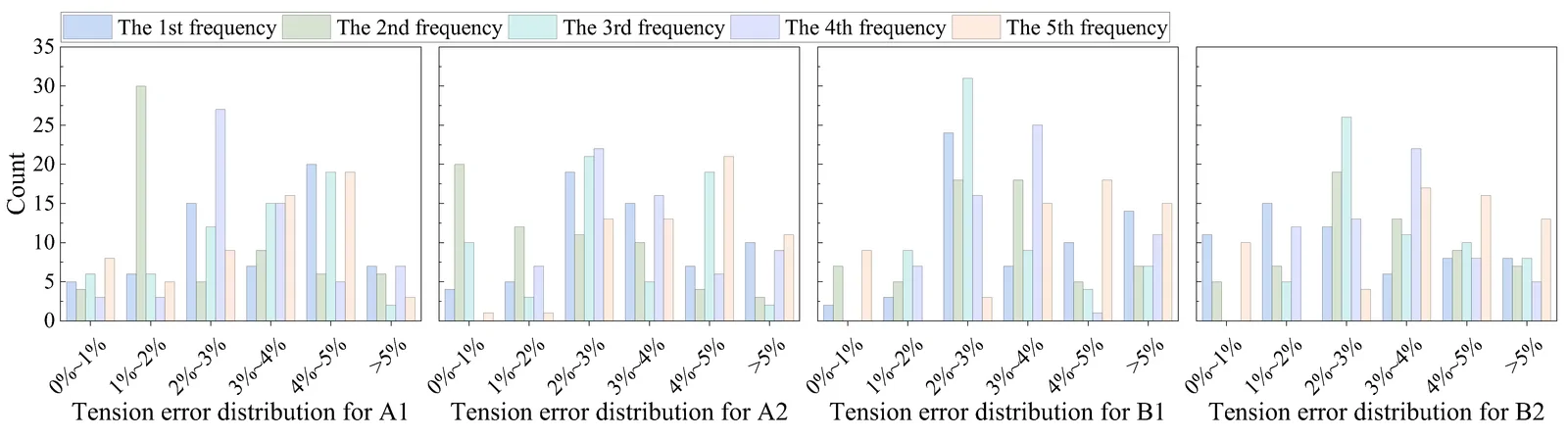
The statistical distribution of the tension errors under four test conditions.

At the same time, the results and errors of the statistical distribution analysis of all 60 independent tests under the four conditions are summarized in Figure 12. The tension errors are categorized into the following ranges: 0–1%, 1–2%, 2–3%, 3–4%, 4–5%, and greater than 5%. For each frequency order, the count of errors within these ranges is reported. Among all results, the majority of errors fall within the range of 1% to 4%, indicating that the proposed method exhibits strong stability and reliability in identifying the natural frequencies and cable tensions. However, a small part of the cases have errors exceeding 5%, primarily associated with higher-order frequency estimates under the B1 test condition. In summary, the proposed method demonstrates consistent and dependable performance in tension identification while maintaining a high level of accuracy. These advantages highlight its potential for the structural health monitoring of the stayed cables in practice.

### 4.3. The Holographic Modal Shapes and Dynamic Displacement Vectors

By leveraging the proposed HFPVM algorithm and the identified natural frequencies, the original motion signal spectral components of the holographic feature points are decoupled and filtered. Subsequently, the morphological motion information corresponding to the specific frequency band of the stayed cables is magnified to facilitate the visualization and extraction of the holographic mode shapes and dynamic displacement vectors.

The test condition A1 is selected as an example; the full-field optical flow of the weak motion signal spectral components is converted over the whole test time history. And then, the motion signal spectral component in a single-frame image is subjected to complex controllable pyramid decomposition. Finally, the motion signal spectral components of the holographic feature point set *P* within the specific frequency bands are selectively amplified. It is worth noting that the motion signal associated with higher-order modes is extremely weak and hard to separate in practice, and forced amplification may lead to distortion of the holographic motion information. Therefore, this study focuses on extracting and visualizing the holographic motion information corresponding to the first three mode components of the stayed cable.

**Figure 13:**
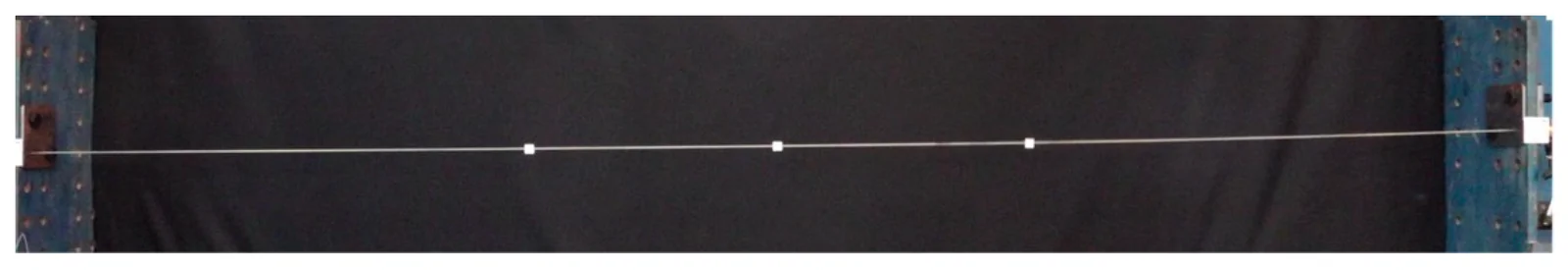
The original image corresponding to a certain frame.

**Figure 14:**
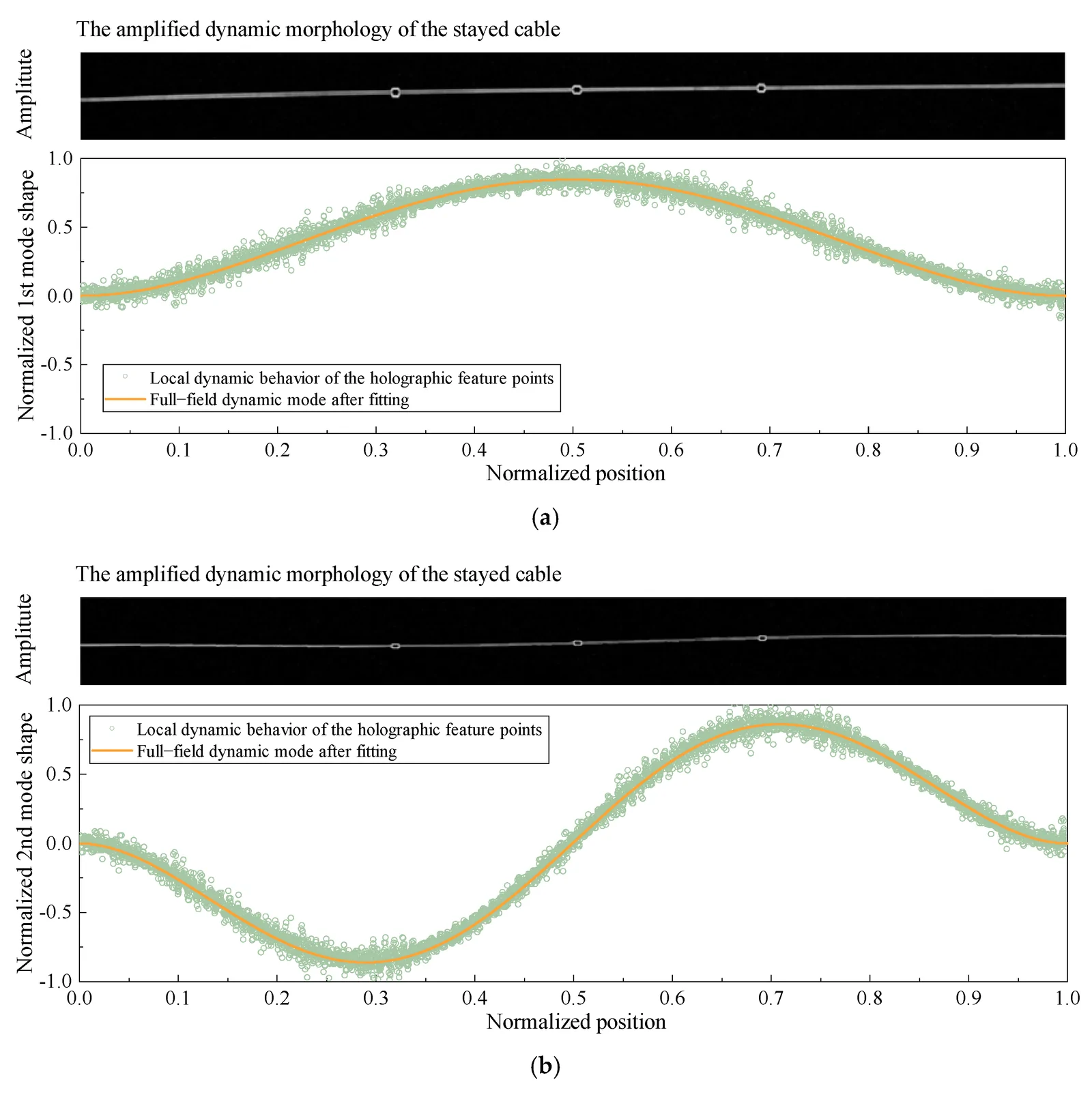

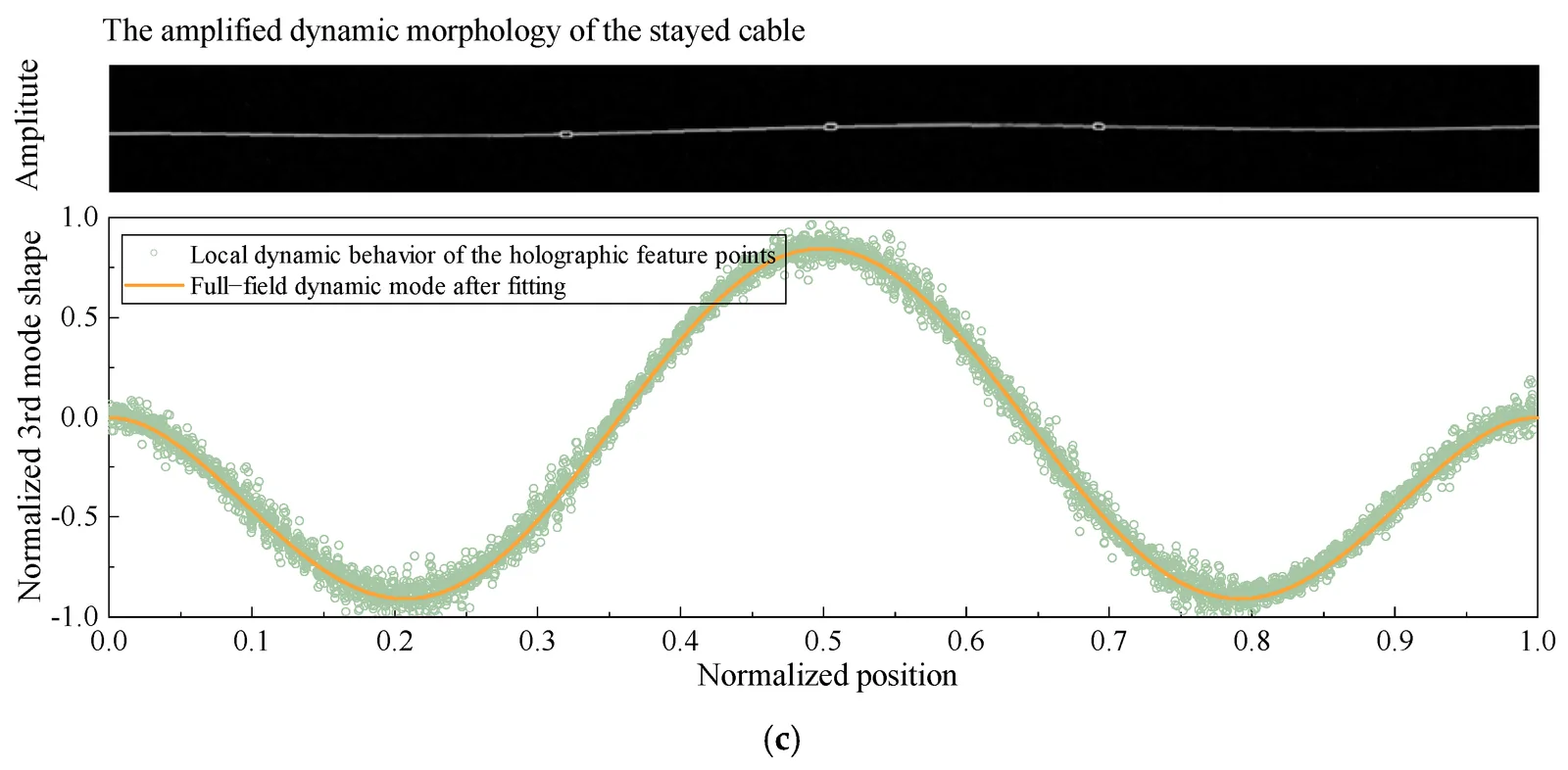
The amplified dynamic morphology and the normalized holographic mode shapes of the stayed cable. (**a**) The first mode. (**b**) The second mode. (**c**) The third mode.

The original image corresponding to a certain frame is shown in Figure 13. Intuitively, due to the slender geometry and low-amplitude vibrations of the stayed cables, the structural deformation is not visually discernible in a certain frame of the original video. After amplifying the specific weak response motion signal of the stayed cable, a complex controllable pyramid reconstruction is performed with the original input image sequence data. The amplified dynamic morphology of the stayed cable and the normalized holographic mode shapes are shown in Figure 14.

From Figure 14, it can be observed that the image signal is enhanced within a specific frequency band. Intuitively, the vibration modes of the stayed cable become visually distinguishable, and the amplified dynamic morphology presents a clear and smooth edge. The motion signal spectral components of the holographic feature point set *P* are also amplified and normalized, and the local dynamic behavior of the holographic feature points is used to fit the full-field dynamic mode of the entire stayed cable. The results of the amplified dynamic morphology and the normalized holographic mode shapes indicate that the proposed HFPVM algorithm in this paper can effectively amplify weak morphological motion information in a specific frequency band and can extract and visualize the modal shapes of stayed cables, providing smoother holographic modal shapes.

The MAC coefficients of the first three normalized holographic mode shapes obtained under different test conditions are shown in Table 6. It is noted that the MAC coefficients are calculated by comparing them with the theoretical values. It is found that the normalized holographic mode shapes have consistently high MAC values across all test conditions. The highest MAC value, 99.51%, was observed in the first-order mode under condition A2, while the lowest value, 97.71%, occurred in the third-order mode under condition B2. Furthermore, a decreasing trend in MAC values is noted with increasing modal order across all test conditions, which can be primarily attributed to the higher susceptibility of higher-order modes to noise and local disturbances. The MAC results confirm that the proposed method can accurately and reliably extract the holographic mode shapes of the stayed cables. In addition, the continuity of the dense holographic feature points in time and space can provide rich dynamic and geometric information, supporting future efforts in structural damage identification.

**Figure 15:**
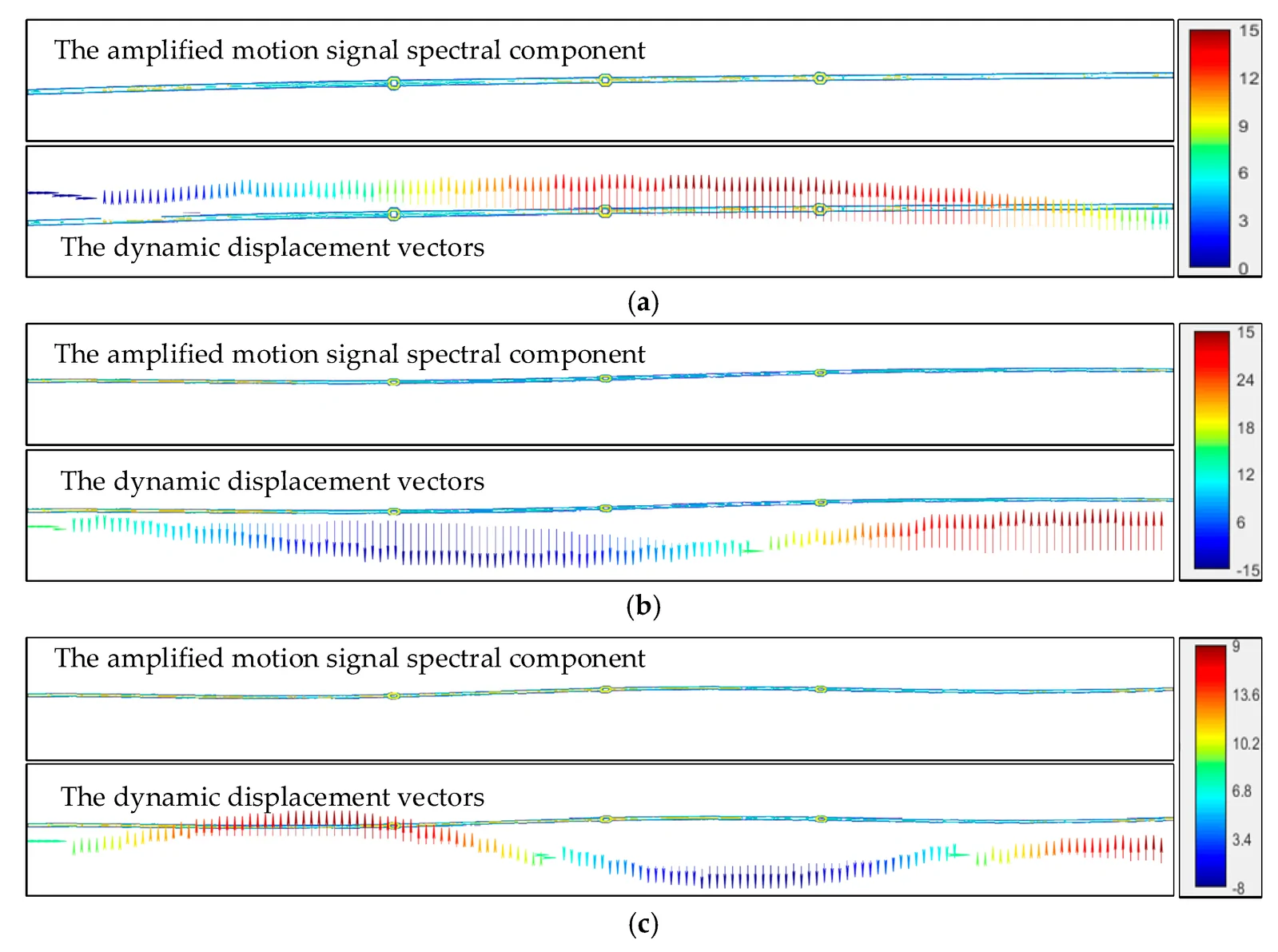
The amplified motion signal spectral component and dynamic displacement vectors. (**a**) Spatial dynamic shape distribution of the first-order component. (**b**) Spatial dynamic shape distribution of the second-order component. (**c**) Spatial dynamic shape distribution of the third-order component.

For a specific time instant, the local dynamic behavior is obtained by sequentially utilizing the holographic feature points based on the corresponding frequency band, phases, and amplitudes. The amplified motion spectral component and the dynamic displacement of the holographic feature points are combined and aligned according to their local dynamic behavior, and finally, the full-field dynamic displacement vector of the stayed cable can be captured. Taking test condition A1 as an example, the amplified spectral component and the dynamic displacement vectors of the first three modes for a specific frame are illustrated in Figure 15. Notably, the dynamic displacement vectors serve as a quantitative and visual representation of the cable’s morphological motion information in space. The dynamic displacement vector serves only as a visualization of a specific dynamic shape of the stayed cable at a particular time instant. It aims to illustrate the spatial dynamic shape distribution of a certain order of motion component; the amplitude and direction of the vectors represent only the relative trend of vibration amplitudes at different positions, rather than absolute physical displacements. The dynamic displacement vector is an essential element in visualizing the dynamic characteristics of stayed cables. As shown in Figure 15, the morphological motion information that is imperceptible in the original image sequence is revealed through the dynamic displacement vectors. The combined use of holographic mode shapes and dynamic displacement vectors effectively captures and visualizes the dynamic behavior of the stayed cable while also preserving the spatiotemporal continuity of the structural response.

### 4.4. Prospect and Discussion

The experimental results demonstrate that the proposed holographic vision-based method performs well in capturing the full-field dynamic parameters and identifying the tension of stayed cables. The identified results show good agreement with those obtained from conventional contact-type sensors. As a non-contact measurement approach, this method achieves, for the first time under laboratory conditions, comprehensive, visualized, and quantifiable monitoring of stayed cables, showing great potential for future applications.

However, when extending the method to real bridge structures, several foreseeable challenges may arise:

**Interference from ambient vibrations**: In real bridge environments, stayed cables are subject to various sources of excitation, such as traffic loads and wind, which often exhibit non-stationary and non-Gaussian characteristics. These environmental disturbances may interfere with the extraction of the cable’s intrinsic modal features, thereby affecting the accuracy of dynamic parameter identification and tension estimation. Therefore, effective noise suppression strategies should be further investigated to improve the stability and reliability of field measurements.

**Influence of lighting conditions**: The visual monitoring performance can be significantly affected by varying lighting conditions on the bridge site, including changing sunlight angles, shadows, and insufficient illumination at night. These factors may degrade image quality and hinder the accurate detection and tracking of holographic feature points, ultimately impairing the extraction of the dynamic displacement.

**Limitations of the shooting distance and the sampling frequency**: In field applications, cameras are often deployed at a distance for safety and full-field coverage, which may reduce image resolution and limit the ability to detect small-amplitude vibrations. Moreover, high-order modal identification demands high frame rates, which may exceed the capabilities of commercial camera systems, leading to loss of high-frequency information. Nevertheless, with the advancement of high-resolution and ultra-high-speed imaging technologies, these physical limitations are expected to be gradually overcome.

**Influence of boundary conditions on tension identification**: In real bridges, the boundary conditions of stayed cables are often far from ideal, and may instead exhibit elastic restraints, frictional interfaces, or nonlinear contact behaviors at the anchorage zones. These non-ideal conditions significantly affect the cable’s dynamic characteristics and, consequently, the accuracy of tension estimation based on modal frequencies.

## 5. Conclusions

This paper proposes a novel holographic vision-based method for enabling the high-order full-field dynamic parameters, i.e., the natural frequencies, damping ratios, holographic modal shapes, dynamic displacement vectors, and cable tensions. This is the first study to propose a comprehensive, visual, and quantifiable strategy for the stayed cable monitoring. A series of laboratory experiments is conducted on the stayed cables under varying installation angles and tension conditions to validate the effectiveness of the proposed method. The main findings are summarized as follows:

(1) The proposed holographic vision-based method demonstrates high accuracy and robustness in identifying the first five natural frequencies of the stayed cables, with errors consistently below 5%, meeting engineering application requirements.
(2) Most damping ratios are also successfully identified with acceptable accuracy; the estimation of higher-order damping ratios (such as the 4th and 5th modes) is occasionally unstable due to signal quality and algorithmic limitations.
(3) The cable tension estimation based on the identified natural frequencies proves to be effective and stable, with a maximum relative error of 6.86%.
(4) The holographic vision-based method allows for accurate extraction and visualization of the first three holographic modal shapes and dynamic displacement vectors. The MAC value reaches up to 99.51%, and the dynamic displacement vectors can also visualize and quantify the imperceptible morphological motion information in the original image sequence.

The proposed holographic vision-based method provides an effective strategy toward realizing comprehensive, full-field monitoring of the stayed cables in real bridge environments. Specifically, the identified full-field dynamic parameters can be used to evaluate the long-term dynamic behavior of the cables and provide early warnings of potential structural anomalies. The estimated cable tensions enable continuous monitoring of cable force stability and facilitate the detection of abnormal tension loss. These capabilities are essential for assessing the safety and serviceability of cable-supported bridge structures throughout their entire service life. By integrating real-time visual data acquisition with digital twin technologies, the method further facilitates the development of intelligent, automated, and data-driven structural health monitoring systems.

## Figures and Tables

**Table 1:** Parametric listing of HVS calibration in the laboratory experiments.

Radial Distortion Coefficient	K1/K2	1.9644 × 10^8^/5.6287 × 10^6^
Eccentric Distortion Coefficient	P1/P2	1.4683 × 10^5^/3.8601 × 10^6^
Pixel Size	0.004096 mm	-
Image Size	8688 × 5792	-
Outline Size	Phase Principal Coordinates α_x_ Phase Principal Coordinates α_y_ Camera Main Distance *f*_c_	−0.0675 mm 0.0042 mm 49.8256 mm

**Table 2:** The technical parameters of the model cable.

Specifications (mm)	Standard Cross-Sectional Area (mm^2^)	Average Elastic Modulus (N/mm^2^)	Nominal Tensile Strength (MPa)	Quality Per Meter (kg/m)
6 mm 7 × 19	14.32	195,000	1570	0.248

**Table 3:** Test conditions and contents.

Test Conditions	Experimental Variables				
	Condition Number	Length-Tilt Angle	Anchor End Height Difference	Cable Tension	Load Duration Time
Horizontal cable	A1	3.5 m-0°	0 cm	~500 N	90 s
	A2	3.5 m-0°	0 cm	~1000 N	90 s
Tilted cable	B1	3.73 m-20.08°	127.9 cm	~700 N	90 s
	B2	3.73 m-20.08°	127.9 cm	~1200 N	90 s

**Table 4:** The multiple test results of the stayed cable model.

Condition	Order	Holographic Vision-Based Method				Accelerometer				Numerical	
		Natural Frequency (Hz)		Damping Ratio (%)		Natural Frequency (Hz)		Damping Ratio (%)		Natural Frequency (Hz)	Damping Ratio (%)
		Average	Error %	Average	Error %	Average	Error %	Average	Error %	Average	Average
A1	1st	6.49	1.9	3.2	3	6.50	2.1	3.2	3	6.37	3.3
	2nd	12.75	0.9	1.8	5.3	12.76	1.0	1.7	11	12.64	1.9
	3rd	18.93	0.8	1.2	9.1	18.95	0.9	1.1	0	18.78	1.1
	4th	25.6	1.5	-	-	25.57	1.4	0.5	16	25.22	0.6
	5th	31.95	2.2	-	-	31.95	2.2	-	-	31.26	0.3
A2	1st	8.95	2.5	4.3	4	8.89	1.8	4.2	6.7	8.73	4.5
	2nd	18.15	2.9	2	13	18.20	3.2	2.2	4.3	17.64	2.3
	3rd	26.84	2.4	1.9	11	26.89	2.6	1.6	5.9	26.21	1.7
	4th	36.33	1.6	0.9	18	37.12	3.8	1.2	9.1	35.76	1.1
	5th	45.38	1.2	-	-	45.25	0.9	-	-	44.84	0.8
B1	1st	7.04	0.2	3.3	15	7.05	0.4	3.6	7.7	7.03	3.9
	2nd	14.09	0.9	2.9	0	14.19	1.6	2.7	6.9	13.96	2.9
	3rd	21.23	0.4	1.8	10	21.25	0.5	1.9	5	21.15	2
	4th	28.44	2.6	1.3	8.3	28.41	2.5	1.1	8.3	27.72	1.2
	5th	35.06	0.8	-	-	35.09	0.9	-	-	34.78	0.6
B2	1st	9.27	3	5.1	1.9	9.68	7.5	4.9	5.8	9.00	5.2
	2nd	18.89	0.4	3.9	5.4	19.98	6.2	3.8	2.7	18.81	3.7
	3rd	27.73	0.4	3	3.5	28.14	1.9	2.6	10	27.62	2.9
	4th	37.95	0.1	-	-	38.75	2.2	1.5	6.3	37.91	1.6
	5th	46.81	0.8	-	-	46.86	0.9	-	-	46.44	0.9

**Table 5:** Average results of the cable tension.

Test Condition	Category	Identified Cable Tension by Equation (23)-N					Average
		1st	2nd	3rd	4th	5th	
A1	Holographic vision-based method	531.73	510.57	496.07	504.82	495.86	507.81
	Relative error	6.67%	2.42%	−0.49%	1.27%	−0.53%	1.87%
	Pressure sensor	498.5					
A2	Holographic vision-based method	992.37	1041.26	1012.02	1042.17	1038.23	1025.21
	Relative error	−0.03%	4.89%	1.95%	4.98%	4.59%	3.27%
	Pressure sensor	992.7					
B1	Holographic vision-based method	705.83	703.24	704.59	704.87	703.08	704.32
	Relative error	1.37%	1.00%	1.19%	1.23%	0.97%	1.15%
	Pressure sensor	696.3					
B2	Holographic vision-based method	1228.80	1274.91	1218.86	1280.66	1241.79	1249.00
	Relative error	2.08%	5.91%	1.25%	6.38%	3.16%	3.76%
	Pressure sensor	1203.8					

**Table 6:** The MAC coefficients of the normalized holographic mode shapes.

Orders	Test Condition	A1	A2	B1	B2
1st	Average frequency, Hz	6.49	8.95	7.04	9.27
	Modal Assurance Criterion, %	99.23	99.51	99.24	98.96
2nd	Average frequency, Hz	12.75	18.15	14.09	18.89
	Modal Assurance Criterion, %	98.52	98.92	98.36	98.47
3rd	Average frequency, Hz	18.93	26.84	21.23	27.73
	Modal Assurance Criterion, %	97.92	98.16	97.85	97.71

## Data Availability

The data presented in this study are available on request from the corresponding author, because the data are not publicly available due to privacy or ethical restrictions.

## References

[1] Ehrhardt D.A., Allen M.S., Yang S., Beberniss T.J. (2017). Full-field linear and nonlinear measurements using Continuous-Scan Laser Doppler Vibrometry and high speed Three-Dimensional Digital Image Correlation. Mech. Syst. Signal Process..

[2] Xu Y.F. (2020). A photogrammetry-based experimental modal analysis method by tracking visible laser spots. Measurement.

[3] Shan B., Zheng S., Ou J. (2015). Free vibration monitoring experiment of a stayed-cable model based on stereovision. Measurement.

[4] Spencer B.F., Hoskere V., Narazaki Y. (2019). Advances in Computer Vision-Based Civil Infrastructure Inspection and Monitoring. Engineering.

[5] Zona A. (2020). Vision-Based Vibration Monitoring of Structures and Infrastructures: An Overview of Recent Applications. Infrastructures.

[6] Shao S., Zhou Z., Deng G., Du P., Jian C., Yu Z. (2020). Experiment of Structural Geometric Morphology Monitoring for Bridges Using Holographic Visual Sensor. Sensors.

[7] Deng G., Zhou Z., Shao S., Chu X., Jian C. (2020). A Novel Dense Full-Field Displacement Monitoring Method Based on Image Sequences and Optical Flow Algorithm. Appl. Sci..

[8] Xu Y., Brownjohn J., Kong D. (2018). A non-contact vision-based system for multipoint displacement monitoring in a cable-stayed footbridge. Struct. Control Health Monit..

[9] Zhao J., Bao Y., Guan Z., Zuo W., Li J., Li H. (2019). Video-based multiscale identification approach for tower vibration of a cable-stayed bridge model under earthquake ground motions. Struct. Control Health Monit..

[10] Dong C.-Z., Celik O., Catbas F.N. (2019). Marker-free monitoring of the grandstand structures and modal identification using computer vision methods. Struct. Health Monit..

[11] Dong C.-Z., Catbas F.N. (2021). A review of computer vision–based structural health monitoring at local and global levels. Struct. Health Monit..

[12] Dong C.-Z., Celik O., Catbas F.N., O’Brien E.J., Taylor S. (2020). Structural displacement monitoring using deep learning-based full field optical flow methods. Struct. Infrastruct. Eng..

[13] Feng D., Scarangello T., Feng M.Q., Ye Q. (2017). Cable tension force estimate using novel noncontact vision-based sensor. Measurement.

[14] Kim S.-W., Jeon B.-G., Kim N.-S., Park J.-C. (2013). Vision-based monitoring system for evaluating cable tensile forces on a cable-stayed bridge. Struct. Health Monit. Int. J..

[15] Zhao X., Ri K., Wang N. (2017). Experimental Verification for Cable Force Estimation Using Handheld Shooting of Smartphones. J. Sens..

[16] Yoon H., Elanwar H., Choi H., Golparvar-Fard M., Spencer B.F. (2016). Target-free approach for vision-based structural system identification using consumer-grade cameras: Target-Free Vision-based Structural System Identification. Struct. Control Health Monit..

[17] Feng D., Feng M.Q. (2017). Experimental validation of cost-effective vision-based structural health monitoring. Mech. Syst. Signal Process..

[18] Dong C.Z., Ye X.W., Jin T. (2018). Identification of structural dynamic characteristics based on machine vision technology. Measurement.

[19] Feng D., Feng M.Q. (2016). Vision-based multipoint displacement measurement for structural health monitoring: Vision-Based Displacement Measurement for SHM. Struct. Control Health Monit..

[20] Ji Y.F., Chang C.C. (2008). Nontarget Stereo Vision Technique for Spatiotemporal Response Measurement of Line-Like Structures. J. Eng. Mech..

[21] Chen J.G., Adams T.M., Sun H., Bell E.S., Büyüköztürk O. (2018). Camera-Based Vibration Measurement of the World War I Memorial Bridge in Portsmouth, New Hampshire. J. Struct. Eng..

[22] Chen J.G., Wadhwa N., Cha Y.-J., Durand F., Freeman W.T., Buyukozturk O. (2015). Modal identification of simple structures with high-speed video using motion magnification. J. Sound Vib..

[23] Yang Y., Dorn C., Mancini T., Talken Z., Kenyon G., Farrar C., Mascareñas D. (2017). Blind identification of full-field vibration modes from video measurements with phase-based video motion magnification. Mech. Syst. Signal Process..

[24] Yang Y., Dorn C., Mancini T., Talken Z., Nagarajaiah S., Kenyon G., Farrar C., Mascareñas D. (2017). Blind identification of full-field vibration modes of output-only structures from uniformly-sampled, possibly temporally-aliased (sub-Nyquist), video measurements. J. Sound Vib..

[25] Fioriti V., Roselli I., Tatì A., Romano R., De Canio G. (2018). Motion Magnification Analysis for structural monitoring of ancient constructions. Measurement.

[26] Tian Y., Zhang J., Yu S. (2019). Vision-based structural scaling factor and flexibility identification through mobile impact testing. Mech. Syst. Signal Process..

[27] Hoskere V., Park J.-W., Yoon H., Spencer B.F. (2019). Vision-Based Modal Survey of Civil Infrastructure Using Unmanned Aerial Vehicles. J. Struct. Eng..

[28] Poozesh P., Baqersad J., Niezrecki C., Avitabile P., Harvey E., Yarala R. (2017). Large-area photogrammetry based testing of wind turbine blades. Mech. Syst. Signal Process..

[29] Srivastava V., Baqersad J. (2019). An optical-based technique to obtain operating deflection shapes of structures with complex geometries. Mech. Syst. Signal Process..

[30] Bharadwaj K., Sheidaei A., Afshar A., Baqersad J. (2019). Full-field strain prediction using mode shapes measured with digital image correlation. Measurement.

[31] Harmanci Y.E., Gülan U., Holzner M., Chatzi E. (2019). A Novel Approach for 3D-Structural Identification through Video Recording: Magnified Tracking. Sensors.

[32] Zhou Z., Shao S., Deng G., Gao Y., Wang S., Chu X. (2021). Vision-based modal parameter identification for bridges using a novel holographic visual sensor. Measurement.

[33] Mehrabi A.B., Tabatabai H. (1998). Unified Finite Difference Formulation for Free Vibration of Cables. J. Struct. Eng..

[34] Zui H., Shinke T., Namita Y. (1996). Practical Formulas for Estimation of Cable Tension by Vibration Method. J. Struct. Eng..

[35] Zappa E., Mazzoleni P., Matinmanesh A. (2014). Uncertainty assessment of digital image correlation method in dynamic applications. Opt. Lasers Eng..

[36] Javh J., Slavič J., Boltežar M. (2017). The subpixel resolution of optical-flow-based modal analysis. Mech. Syst. Signal Process..

[37] Horn B.K.P., Schunck B.G. (1981). Determining optical flow. Artif. Intell..

[38] Lee J., Shin S.Y. (2002). General construction of time-domain filters for orientation data. IEEE Trans. Vis. Comput. Graph..

[39] Karasaridis A., Simoncelli E. A filter design technique for steerable pyramid image transforms. Proceedings of the 1996 IEEE International Conference on Acoustics Speech, and Signal Processing Conference Proceedings.

[40] Wu H.-Y., Rubinstein M., Shih E., Guttag J., Durand F., Freeman W.T. (2012). Eulerian Video Magnification for Revealing Subtle Changes in the World. ACM Trans. Graph. TOG.

[41] Sushma M., Gupta A., Sivaswamy J., Maji P., Ghosh A., Murty M.N., Ghosh K., Pal S.K. (2013). Semi-automated Magnification of Small Motions in Videos. Proceedings of the Pattern Recognition and Machine Intelligence.

[42] Wu X., Yang X., Jin J., Yang Z. (2018). Amplitude-Based Filtering for Video Magnification in Presence of Large Motion. Sensors.

[43] Wu X., Yang X., Jin J., Yang Z. (2018). PCA-based magnification method for revealing small signals in video. Signal Image Video Process..

[44] Fukuda Y., Feng M.Q., Narita Y., Kaneko S., Tanaka T. (2013). Vision-Based Displacement Sensor for Monitoring Dynamic Response Using Robust Object Search Algorithm. IEEE Sens. J..

[45] Caetano E. (2007). Cable Vibrations in Cable-Stayed Bridges.

[46] Zhou K., Li Q.-S., Han X.-L. (2022). Modal Identification of Civil Structures via Stochastic Subspace Algorithm with Monte Carlo–Based Stabilization Diagram. J. Struct. Eng..

[47] Yang X.-M., Yi T.-H., Qu C.-X., Li H.-N., Liu H. (2019). Automated Eigensystem Realization Algorithm for Operational Modal Identification of Bridge Structures. J. Aerosp. Eng..

